# A new regulator of the *Staphylococcus aureus* peptidoglycan hydrolase Sle1

**DOI:** 10.1371/journal.pgen.1011990

**Published:** 2025-12-31

**Authors:** Helena Veiga, Adrian Izquierdo-Martinez, Leonor B. Marques, Mariana G. Pinho

**Affiliations:** Instituto de Tecnologia Química e Biológica António Xavier, Universidade Nova de Lisboa, Oeiras, Portugal; Tufts University School of Medicine, UNITED STATES OF AMERICA

## Abstract

Regulation of peptidoglycan hydrolases is crucial for bacterial cell integrity, growth and division. In the bacterial pathogen *Staphylococcus aureus*, the amidase Sle1 is a key autolysin required for septum splitting and daughter cell separation. Through genetic suppressor screening, we have identified CxaR, a previously uncharacterized protein, as a novel negative regulator of Sle1. In the absence of CxaR, cellular levels of Sle1 increase nearly ten-fold, resulting in premature splitting of the division septum and increased cell lysis during exponential growth. CxaR localizes to the division septum, late in septum synthesis, and this localization requires both the divisome protein FtsK and the ClpX component of the ClpXP proteolytic machinery. We propose that CxaR promotes ClpXP-mediated degradation of Sle1 towards the end of the cell cycle.

## Author summary

The final step in cell division of the major pathogen *Staphylococcus aureus* is the separation of the two newly formed daughter cells, a process that relies on the autolysin Sle1 to cleave the cell-wall layer between them. Because this layer is essential for maintaining cell integrity, Sle1’s activity must be tightly controlled. If Sle1 acts at the wrong time, it can breach the dividing wall too early, weakening the bacteria and potentially increasing their vulnerability to host defenses and lysis. Understanding how this enzyme is regulated is likely to enable new strategies for harnessing Sle1 as a lysis agent to more effectively combat *S. aureus* infections in the future. In this study, we identified a previously unknown regulator of Sle1, CxaR. The absence of CxaR leads to a nearly ten-fold increase in Sle1 levels, causing premature septum splitting and making cells more prone to lysis during growth. We further found that CxaR accumulates at the division site late in the cell cycle, a process that requires the divisome protein FtsK and the protease adaptor ClpX. Together, these findings support a model in which CxaR directs Sle1 to the ClpXP protease for controlled degradation.

## Introduction

Most bacterial cells are surrounded by a cell wall that confers shape and resistance to internal turgor pressure. The main component of the cell wall is peptidoglycan, a large molecule composed of glycan strands cross-linked by flexible peptides [1]. The importance of peptidoglycan to bacterial cell survival is demonstrated by the effectiveness and wide use of various classes of antibiotics, such as beta-lactams or glycopeptides, which target its synthesis [2]. Given the relevance of peptidoglycan synthases as targets for some of the most widely used antibiotics, these enzymes have been extensively studied for over five decades. However, bacterial growth and division require the concerted action not only of peptidoglycan synthases but also of peptidoglycan hydrolases [3,4]. Endogenous peptidoglycan hydrolases (also known as autolysins) are required for the insertion of new peptidoglycan material and the expansion of the cell surface during growth, as well as for splitting the division septum at the end of the cell cycle to separate the mother cell into two daughter cells [4,5]. Although the activity of peptidoglycan hydrolases is crucial for bacterial growth, it must be tightly regulated to prevent cell lysis [5–7].

The gram-positive bacterial pathogen *Staphylococcus aureus* has at least 18 different autolysins with distinct activities, enabling the cleavage of virtually any bond in its peptidoglycan [8]. Among these enzymes are amidases, such as Atl, Sle1 or LytN, which cleave the bond between the N-acetyl muramic acid in the glycan strands and L-Ala, the first amino acid of the stem peptide [9–11]. These enzymes are critical for septum splitting, which is required for cell separation, and therefore their absence impairs normal cell cycle progression [12].

*S. aureus* cells synthesize a complete septum before initiating septum splitting, which in this bacterium is an extremely fast process, taking less than two milliseconds [12,13]. This remarkable speed implies that the septum material, which will become future cell surface after splitting and remodelling, must undergo maturation prior to the separation of the daughter cells. Otherwise, an immature cell wall, lacking capsular polysaccharide or multiple surface proteins that act as virulence factors, would be exposed to the exterior, potentially facilitating clearance of bacterial cells by the infected host [14,15].

Consistent with its critical role in septum splitting, Sle1 is regulated by multiple mechanisms that operate at different stages the protein’s life cycle. Transcription of Sle1 is under the control of the two-component systems WalKR, which controls the expression of various autolysin-encoding genes [16], and GraSR, involved in glycopeptides and cationic antimicrobial peptides resistance [17,18]. Furthermore, abundance of the Sle1 protein is controlled through regulated degradation by ClpXP, a proteolytic machinery responsible for degrading damaged or unneeded proteins in the cell [19–21]. Besides the precise regulation of its protein levels, Sle1 localization is also strictly controlled. Sle1 primarily localizes to the division septum, in agreement with its role in the separation of daughter cells [22,23]. Two mechanisms have been proposed to mediate Sle1 septal localization. One is based on the binding of Sle1’s LysM domains to peptidoglycan, which is prevented by the presence of wall teichoic acids [23]. These are ribitol-phosphate polymers that are tethered to the N-acetyl muramic acid residues of the peptidoglycan. Since mature wall teichoic acids are present at the cell periphery but mostly absent at the septum, a purified fluorescent derivative of Sle1’s LysM domains was found to accumulate at the septal region of *S. aureus* cells [23]. A second mechanism to control the localisation of endogenously-produced Sle1 involves the formation of a complex between Sle1, the chaperone Trigger Factor, and the divisome protein FtsK, which localizes at the septum [22]. Notably, FtsK regulates not only the localization but also abundance of Sle1, as in the absence of this divisome protein, Sle1 is completely degraded by ClpXP and becomes undetectable in *S. aureus* cells [22].

Given the intricate regulation of Sle1, we wondered if additional mechanisms remained to be identified. To find novel regulators of Sle1, we performed a genetic screening for suppressors that restore Sle1 abundance in the absence of FtsK, leading to the identification of SACOL0710 as a negative regulator of this autolysin.

## Results

### Identification of CxaR as a regulator of Sle1

We previously screened for regulators of Sle1 by constructing a translational fusion between this autolysin and the *S. aureus* PhoB alkaline phosphatase [22]. PhoB is only active on the outer surface of the cell [24] and its activity can be detected using the chromogenic alkaline phosphatase substrate 5-Bromo-4-chloro-3-indolyl phosphate (BCIP) (Fig A in S1 Text). Given that Sle1 is present on the outer surface of the cell, expression of the Sle1-PhoB fusion in an *S. aureus* strain lacking its native PhoB results in the restoration of blue colonies in the presence of BCIP [22]. This reporter strain, when used to identify mutants lacking surface localized Sle1-PhoB (that appear as white colonies), led to the identification of FtsK as a factor required for the presence of Sle1 in *S. aureus* cells [22]. We reasoned that performing transposon mutagenesis in the background of strain COLΔ*phoB* Sle1-PhoB Δ*ftsK*, which produces white colonies in the presence of BCIP, and screening for mutants that produce blue colonies, could lead to the identification of negative regulators of Sle1 (Fig A in S1 Text). Screening of 96000 colonies identified 34 dark blue colonies, including 13 with a transposon insertion in *clpX* and 5 in *clpP.* This was expected, as lack of Sle1 in an FtsK mutant is due to ClpXP mediated degradation of this autolysin [22]. Interestingly, 16 additional blue colonies had a transposon insertion in a previously uncharacterized gene, SACOL0710.

While preparing this manuscript, Barbuti and colleagues reported on a protein encoded by the same gene [25]. To avoid ambiguity in the literature, we have adopted the same nomenclature and will refer to SACOL0710 as CxaR [25]. CxaR is a 165 amino acid protein predicted to be cytoplasmic and previously reported to be downregulated approximately twofold in a *vraTSR* mutant and upregulated 1.5- to 3-fold in response to oxacillin, daptomycin, antimicrobial peptides or brilacidin (which causes membrane depolarization) [26–28].

**Fig 1:**
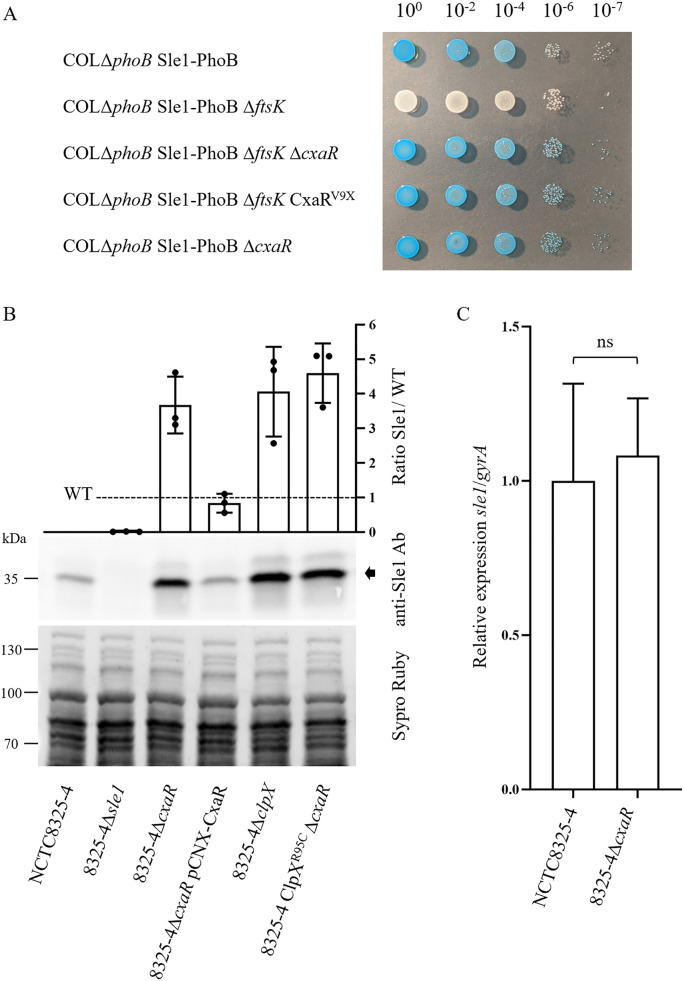
CxaR negatively regulates Sle1 protein levels. **(A)** Serial dilutions of overnight cultures (from 10^0^ to 10⁻^7^) of the indicated strains, plated on TSA containing BCIP, a chromogenic substrate used to detect activity of the alkaline phosphatase PhoB. In this system, blue colonies indicate the presence of Sle1-PhoB fusion on the outer surface of the bacterial cells. Colonies become white upon inactivation of *ftsK*, as previously reported [22]. Deletion of *cxaR* in strain COLΔ*phoB* Sle1-PhoB ∆*ftsK* ∆*cxaR* or introduction of a premature stop codon in strain COLΔ*phoB* Sle1-PhoB ∆*ftsK* CxaR^V9X^ reinstates Sle1-PhoB at the cell surface, leading to blue colonies. **(B)** Quantification of total Sle1 levels by Western blot, using an anti-Sle1 antibody, of extracts of indicated strains. A representative blot is shown below, including the Sypro Ruby staining used for sample normalization; the arrow indicates the Sle1 band. Increased Sle1 levels were observed upon deletion on *cxaR* or of the *clpX* gene, which encodes one of the components of the ClpXP proteolytic machinery*.* Strain 8325-4Δ*cxaR* pCNX-CxaR was grown in the presence of 1 µM cadmium chloride. The graph shows the ratio of Sle1 levels in each strain relative to the parental strain NCTC8325-4 (dashed line). Data are presented as a bar with scatter plot with mean ± SD (n = 3 biological replicates). **(C)** Relative *sle1* mRNA levels in parental strain NCTC8325-4 and *cxaR* deletion mutant 8325-4Δ*cxaR* determined by qPCR, showing that *cxaR* deletion did not affect *sle1* transcription. Expression values were normalized to the housekeeping gene *gyrA* and calculated as fold change relative to the wild-type average. Data represent three independent biological replicates per strain. Statistical significance was determined using a two-tailed Mann–Whitney U test; *P* > 0.05 was considered not significant (ns).

To confirm the results from the screening, specifically that the restored blue color in strain COLΔ*phoB* Sle1-PhoB Δ*ftsK* upon transposon inactivation of *cxaR* was indeed caused by the loss of a functional *cxaR* gene, we constructed a new mutant strain (COLΔ*phoB* Sle1-PhoB Δ*ftsK* Δ*cxaR*) by deleting *cxaR* from the original screening strain. As expected, deletion *cxaR* converted the white colonies into blue (Fig 1A). Furthermore, to minimize potential polar effects caused by the complete deletion of *cxaR*, we constructed a second mutant strain, COLΔ*phoB* Sle1-PhoB ∆*ftsK* CxaR^V9X^, where *cxaR* was inactivated by the introduction of a premature stop codon. This mutation also restored the blue color that indicates expression of Sle1-PhoB (Fig 1A), confirming that the inactivation of CxaR leads to the reappearance of Sle1-PhoB at the surface of an FtsK mutant.

These results suggest that CxaR is a negative regulator of Sle1 and, therefore, that cellular levels of Sle1 should increase in the absence of CxaR. To confirm this, we deleted the gene encoding CxaR in the background of methicillin susceptible *S. aureus* (MSSA) strain NCTC8325-4 (8325-4Δ*cxaR*) and methicillin resistant *S. aureus* (MRSA) strain JE2 (JE2Δ*cxaR*). Western blot analysis using an anti-Sle1 antibody (Fig 1B and Fig B panel A in S1 Text) showed a 3.7 ± 0.8-fold increase in Sle1 levels in 8325-4Δ*cxaR* (that revert to wild type levels upon complementation with plasmid encoded CxaR), and a 10 ± 1.7-fold increase in JE2Δ*cxaR.* These levels are similar to the high Sle1 levels detected upon deletion of *clpX,* which abolishes proteolytic degradation of Sle1 by ClpXP (Fig 1B) [19,20,22]. Proteomic analysis of JE2Δ*cxaR* further confirmed the role of CxaR in Sle1 regulation, as Sle1 levels increased nine-fold in the absence of CxaR compared to the parental strain JE2 (Table A in S1 Text). Importantly, we confirmed that CxaR-mediated regulation of Sle1 was post-translational, as qPCR showed that *sle1* transcript levels do not vary upon deletion of *cxaR* (Fig 1C). This post-translational regulation of Sle1 levels by CxaR is not dependent on decreased levels of the ClpXP proteolytic machinery, as these are maintained in the absence of CxaR (Fig C in S1 Text).

### Lack of CxaR results in cell lysis and premature septum splitting

**Fig 2:**
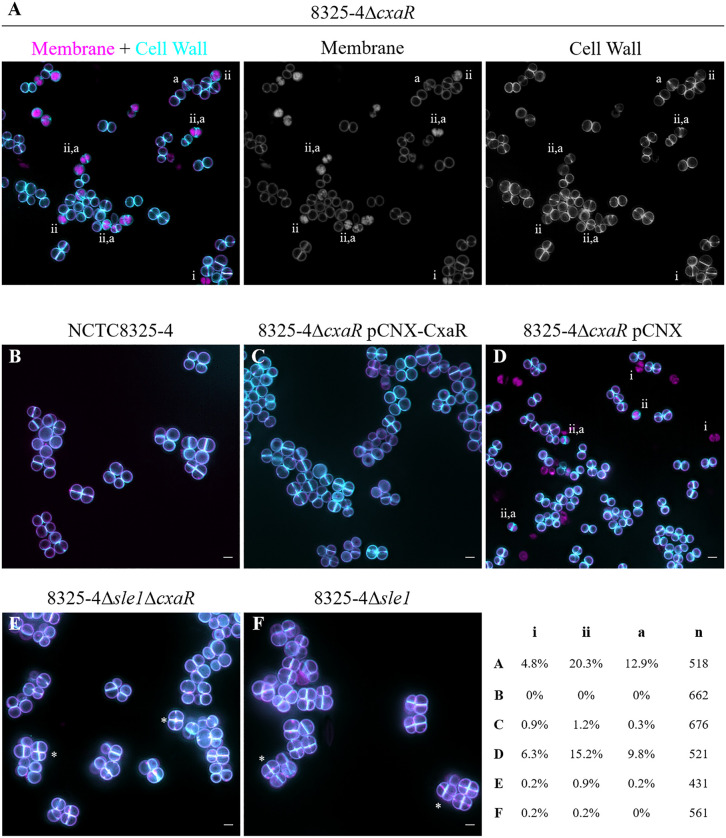
Absence of CxaR leads to premature septum splitting and cell lysis. **(A)** SIM images of the 8325-4Δ*cxaR* mutant labelled with the membrane dye Nile Red (middle) and the fluorescent D-amino acid HADA, which incorporates into the cell wall during growth (right), and an overlay of the two channels (left; cell wall signal in cyan, membrane signal in magenta). **(B–F)** Overlay SIM images of membrane- and cell-wall–stained cells of the indicated strains (individual channels shown in Fig D in S1 Text). Representative images from at least 3 biological replicates. Scale bars 1 µm. In panels A and D, the phenotypes characteristic of the Δ*cxaR* mutant are indicated, and their frequencies are summarized in the accompanying table (n = total number of cells analysed): (i) lysed cells which did not incorporate HADA, (ii) lysed cells labelled with both dyes, and (a) cells where septum splitting occurs prematurely, before septum synthesis is completed, some of which are lysed. These phenotypes are virtually absent in the parental strain NCTC8325-4 **(B)** and in the ∆*cxaR* mutant complemented with plasmid encoded *cxaR* **(C)** but persist in the strain carrying the empty pCNX vector **(D)**. In the 8325-4Δ*sle1*Δ*cxaR* strain **(E)**, similarly to the 8325-4Δ*sle1* mutant **(F)**, tetrads characteristic of *sle1* deletion can be observed (asterisks). Strains 8325-4Δ*cxaR* pCNX and 8325-4Δ*cxaR* pCNX-CxaR were grown in the presence of 1 µM cadmium chloride.

When growing strain JE2Δ*cxaR,* we noticed an initial increase in OD, followed by a decrease that could suggest cell lysis, before growth resumes (Fig B panel B in S1 Text). This was confirmed when Δ*cxaR* cells were observed under the microscope, as lysed cells were frequently observed (Fig 2A, Fig B panel C in S1 Text). Labelling Δ*cxaR* cells with membrane dye Nile Red and fluorescent D-amino acid HADA, which was incorporated into the cell wall during growth (37°C for 30 minutes), allowed visualization of lysed cells which were metabolically inactive upon labelling and therefore did not incorporate HADA (cells labelled (i) in Fig 2A and Fig B panel C in S1 Text), as well as lysed cells that were labelled with both dyes but showed aberrant membrane signal (cells labelled (ii) in Fig 2A and Fig B panel C in S1 Text). Furthermore, Δ*cxaR* mutants showed cells where septum splitting occurred prematurely, i.e., was initiated before septum synthesis was completed (cells labelled (a) in Fig 2A and Fig B panel C in S1 Text, some of which were lysed), a phenotype that was not observed in wild type *S. aureus* cells (Fig 2B and Fig D panel B in S1 Text). Importantly, cell lysis and premature septum splitting virtually disappeared when the Δ*cxaR* mutant was complemented with plasmid encoded *cxaR* in strain 8325-4∆*cxaR* pCNX-CxaR (Fig 2C and Fig D panel C in S1 Text), but not with empty pCNX plasmid (Fig 2D and Fig D panel D in S1 Text). These phenotypes are likely due to an excess of Sle1, as they were abolished upon deletion of both *sle1* and *cxaR* in strain 8325-4Δ*sle1* ∆*cxaR* (Fig 2E and Fig D panel E in S1 Text). In contrast, this strain shows some tetrads of cells, where a second round of division is initiated before the previous septum is split, a phenotype characteristic of Sle1 mutants (Fig 2F and Fig D panel F in S1 Text) [22]. Together these results show that lack of CxaR results in increased Sle1 levels which are deleterious for *S. aureus* cells.

### CxaR localizes to the division septum

**Fig 3:**
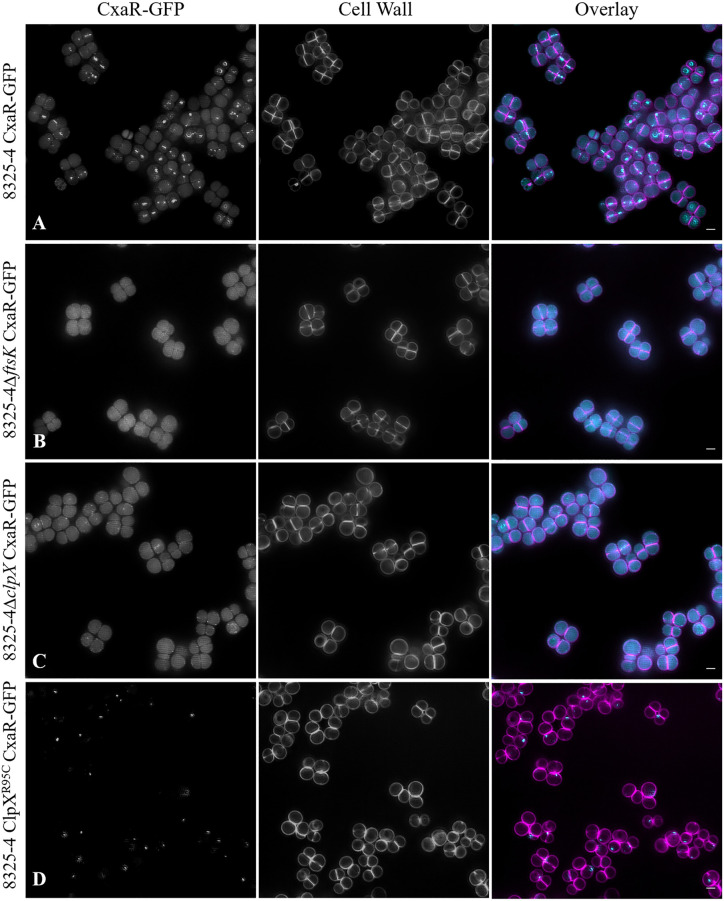
CxaR localizes to the centre of the septum in a fraction of *S. aureus* cells. **(A)**. Structured Illumination Microscopy (SIM) images of 8325-4 CxaR-GFP expressing a fluorescent derivative of CxaR from *cxaR* native chromosomal locus as the sole source of CxaR (left), labelled with fluorescent D-amino acid HADA which is incorporated into the cell wall (middle) and overlay of the two channels (right; CxaR-GFP signal in cyan, cell wall signal in magenta), showing late septal localization of CxaR-GFP. **(B-D)** SIM images showing CxaR-GFP localization upon deletion of *ftsK* **(B)**, deletion of *clpX* **(C)** or inactivation of ClpX **(D)**. CxaR-GFP septal localization is lost in the absence of FtsK or ClpX, while the protein accumulates in foci upon inactivation of ClpX. Representative images from 3 biological replicates. Scale bars 1 μm.

To determine the localization of CxaR, we constructed strain 8325-4 CxaR-GFP which expresses a GFP fluorescent derivative of CxaR from the native locus, as the single copy of *cxaR* in the cell. This strain showed the presence of 7.9% (n = 470) of cells in tetrads (Fig 3A) suggestive of decreased Sle1 levels (8325-4∆*sle1* shows 9.3% of tetrads, n = 626, Fig 2, similarly to what was previously described [22]). This was confirmed by western blot analysis (Fig E in S1 Text) indicating that the CxaR-GFP fusion maybe hyperfunctional, perhaps due to increased stability. CxaR-GFP localizes as focus/small ring at the division septum in a fraction of the cells (Fig 3A). This could be due to (i) CxaR accumulating at the septum as a response to an unknown signal/insult only in a fraction of the cells or to (ii) CxaR being produced only during a specific stage of the cell cycle or at low levels that become detectable by fluorescence microscopy only when concentrated in a small region of the cell during a brief period. To distinguish between these hypotheses, we performed time lapse experiments and found that in the first image of the time lapse only a fraction of cells (37%, n = 275) showed localized CxaR-GFP (Fig F in S1 Text). However, over the course of the timelapse (1 hour in M9 medium, at 37°C) CxaR foci appeared in the vast majority of cells (87%, n = 275) (Fig F in S1 Text). Notice that we did not image the complete cell cycle of every cell and that photobleaching may result in loss of signal in some cells, likely leading to an underestimation of the number of cells with a focus over the course of the experiment. Therefore, we conclude that CxaR is likely to form foci in every cell, but not during the entire cell cycle.

In the cells where CxaR was detected, it localized to the division septum. However, unlike most septal proteins previously studied in *S. aureus,* which localize to midcell early in septum formation, CxaR was observed mainly at the center of the septum and not at the periphery. This suggests that CxaR does not arrive to the septum at the onset of synthesis, but rather at a later stage. To investigate the mechanism of its localization, we tested whether CxaR septal localization was dependent on (i) FtsK, given that CxaR was identified as a suppressor of Sle1 absence in an FtsK mutant or (ii) ClpX, given the similarities between the cell lysis and premature septum splitting phenotypes of CxaR mutants and those observed in ClpXP mutants, as well as the fact that Sle1 proteolysis is mediated by ClpXP [19,20,22,29]. Deletion of either *ftsK* or *clpX* resulted in loss of the bright septal CxaR foci (Fig 3B, 3C), which was not due to loss of CxaR-GFP production (Fig G in S1 Text). Interestingly, when we determined CxaR localization in strain 8325-4 ClpX^R95C^ CxaR-GFP, which carries the inactive R95C allele of ClpX [22], the localization pattern was strikingly different from that observed in the absence of ClpX, with a large fraction of cells displaying CxaR in foci (Fig 3D). This could indicate that CxaR interacts with ClpX, accumulating at the ClpXP proteolytic machinery when this is inactive. Supporting this interpretation, we detected an interaction between ClpX and CxaR using a bacterial two hybrid assay [30] (Fig H in S1 Text). This suggests that CxaR may function as an adaptor protein, facilitating Sle1 degradation by ClpXP, as recently proposed by Barbuti and colleagues [25]. We were unable to detect an interaction between CxaR and Sle1 using the bacterial two hybrid assay, but a negative result in this assay does not exclude the possibility that such an interaction occurs in *S. aureus* cells. Consistent with the hypothesis that CxaR regulates Sle1 through ClpXP-mediated degradation, Sle1 levels in strain 8325-4 ClpX^R95C^ Δ*cxaR*, a mutant lacking *cxaR* and carrying an inactive allele of *clpX,* were similar to those observed in strains 8325-4Δ*cxaR* and 8325-4Δ*clpX*, which lack CxaR or ClpX, respectively (Fig 1B). This indicates that there is no additive or synergistic effect from the absence of both proteins, as expected if they act within the same regulatory pathway.

### CxaR has no homologues of known function

**Fig 4:**
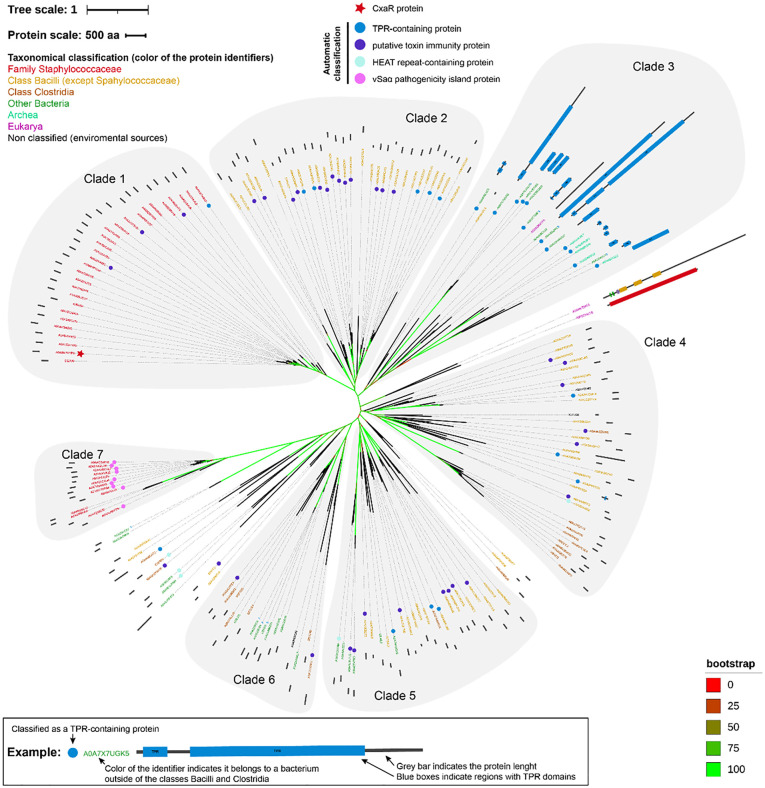
Phylogenetic tree of potential homologs of the CxaR protein. Unrooted tree based on a maximum likelihood analysis of protein sequences from CxaR and 179 potential homologs. Bootstrap support values are colour coded according to the scale on the bottom right corner. Tree scale (top left corner) indicates substitutions per site. Each leaf contains, from inside to outside: (1) a small symbol indicating either the CxaR protein (red star) or the Uniprot automated classification of each protein (coloured circles), except for unclassified proteins or those belonging to classes represented by fewer than three proteins in the tree; (2) the Uniprot accession number for each protein sequence, colour-coded according to the taxonomic classification of the organism it belongs to (upper left corner); and (3) a schematic representation of protein length (dark grey bar) and domains identified by a scan using PROSITE (blue: TRP domain regions; green: EGF-like domains; purple: BTPI domains; yellow: VWFD domains; red: FAS1 domains). The scale bar for protein length is shown in the top left corner. A representative example of leaf annotation is shown at the bottom of the figure. Relevant tree clades (numbered 1-7) are highlighted with a light grey encompassing the corresponding branches and leaves.

To investigate the presence of CxaR homologs in other species, we first used BLAST [31] webserver to find closely related homologs. We found hits with proteins from bacteria from the genus *Staphylococcus* and other members of the Staphylococcaceae family (genera *Macrococcus* and *Mammaliicoccus*), but also from other members of the Bacilli class, including *Bacillus cereus*, *Bacillus thuringiensis*, *Lysinibacillus mangiferihumi* or *Rossellomorea aquimaris,* among others. In order to gain more insights into this family of proteins, we used these initial hits to create a Hidden Markov Model (HMM) of the CxaR proteins to search for more homologs in the Uniprot database [32]. After discarding highly redundant sequences, we obtained a dataset that contained 180 hits. Although most proteins belonged to organisms from the Bacillota phylum (Firmicutes), we also found proteins from more distant organisms, including Fusobacteriota, the FCB group and even a few archaea and eukaryotes. To explore the relationships between the sequences in the dataset, we generated a phylogenetic maximum likelihood tree (Fig 4), where CxaR is in Clade 1, together with other sequences (mostly annotated as of unknown function) from members of the Staphylococcaceae family. Clade 2 is a sister group with Clade 1 and contains sequences from other members of the Bacilli class, most sequences of which are annotated either as putative immunity protein 30 (Imm30) homologs [33] or tetratricopeptide (TPR)-containing proteins. Clade 3 (sister group of clades 1 and 2) is mostly composed of sequences from bacteria outside of the Firmicutes, as well as a few archaea, with most sequences annotated as TPR-containing proteins. The rest of the sequences are more distant to CxaR, most are from Firmicutes (except clade 6) and several are again annotated as immunity proteins or TPR-containing proteins. Interestingly, the distal clade 7 contains sequences exclusively from members of the Staphylococcaceae and most of the sequences are annotated as part of the pathogenicity island νSaα [34]. The genes coding for these proteins have no known function and are located near the *guaA* gene that marks the start of the νSaα pathogenicity island.

Collectively, this analysis reveals that CxaR is related to putative TPR-containing proteins and immunity proteins that are found in many bacterial species. Interestingly, the basic function of TPR domains is to mediate protein-protein interactions, often facilitating the assembly of multiprotein complexes [35,36]. Immunity proteins are also known to engage in protein-protein interactions as immunity proteins without catalytic activity tend to bind to a cognate toxin or a toxin target to prevent the toxin’s activity [37]. This could further support the hypothesis that CxaR activity requires direct protein-protein interaction, most likely with ClpX and Sle1.

## Discussion

The activity of peptidoglycan hydrolases must be tightly regulated to prevent cell lysis or premature splitting of the septal wall at the end of the cell cycle. In this work, we identified CxaR, a previously uncharacterized protein, as a regulator of the *S. aureus* septum-splitting amidase Sle1. Deletion of *cxaR* results in up to ten-fold increase in cellular levels of Sle1. These unregulated levels lead to increased cell lysis during the exponential growth phase and to cells in which splitting of the peripheral region of the septum is initiated before septum synthesis is complete. This premature septum splitting is likely detrimental, as it may expose immature cell wall to the external environment or the host during infection, and contribute to subsequent lysis. Our data are in line with results from Barbuti and colleagues [25], who independently identified CxaR as a negative regulator of Sle1 during a genome-wide screen for determinants of penicillin G susceptibility in *S. aureus* and showed that absence of CxaR leads to growth defects, premature cell splitting and increased cell lysis.

Interestingly, the ORF encoding CxaR is upregulated in the presence of cell wall targeting compounds such as oxacillin, daptomycin or antimicrobial peptides [26–28], suggesting that it may be part of the cellular response to disruptions in cell envelope homeostasis, promoting Sle1 degradation when cell envelope integrity is compromised.

Sle1 is a well-established substrate for the ClpXP proteolytic machinery [19,20,22]. ClpXP substrates can be directly targeted to the proteolytic machinery or may require adaptor proteins that recognize specific substrates and direct them for ClpXP-mediated degradation [38–41]. We have shown that in the absence of CxaR, ClpXP-mediated degradation of Sle1, which eliminates Sle1 from *S. aureus* cells lacking FtsK, is abolished. Furthermore, we have some evidence that CxaR interacts with ClpX, from the bacterial two hybrid assay performed and from the fact that CxaR is sequestered in foci in cells with an inactive ClpX. This is agreement with the recent report by Barbuti and colleagues, who used a split luciferase assay to show that CxaR interacts with both ClpX and Sle1 and suggest that CxaR is an adaptor protein that promotes ClpXP-mediated degradation of Sle1 [25].

Interestingly, CxaR localized to the division septum, becoming concentrated at the inner region of the septum at late stages of septum synthesis, suggesting that its activity is only required temporarily and that Sle1 degradation is under strict temporal control over the course of the cell cycle. This is in line with the fact that Sle1 is involved in septum splitting, the last step of the cell cycle before the mother cell is split in two daughter cells. We have previously shown that Sle1 is exported preferentially at the septum to where it is recruited through the formation of tripartite complex including FtsK, the chaperone Trigger Factor and Sle1 [22]. It is possible that when sufficient levels of Sle1 have been exported to the septal wall (where Sle1 can no longer be degraded by the cytoplasmic ClpXP proteolytic machinery), the cell initiates targeted degradation of this peptidoglycan hydrolase, mediated by septal accumulated CxaR.

The mechanism for CxaR localization at the septum, however, is not completely clear. We showed that CxaR loses its septal localization in the absence of either FtsK or ClpX. This could indicate that CxaR requires both proteins simultaneously for septal localization, or that its localization depends on ClpX, which in turn would be dependent of the presence of FtsK. ClpX has been previously shown to localize in foci near the septum [42], a pattern that we have also observed (Fig I in S1 Text). However, if CxaR localizes at the septum, where it can be observed as small rings as the septum closes (Fig 3A and Fig F in S1 Text), and if it interacts with ClpX, then one would expect ClpX to exhibit a similar septal localization during at least part of the cell cycle. Similarly to *S. aureus,* ClpX is also typically localized in a single bright focus in *E. coli*, but this localization was shown to be an artifact caused by the use of fluorescent tags [43]. When truly monomeric fluorescent proteins were used, ClpX and ClpP displayed cytoplasmic fluorescence in *E. coli* [43]. It is therefore possible that ClpX foci in *S. aureus* are also artifacts. It will be interesting to determine whether *S. aureus* ClpX colocalizes with the closing septum, similarly to CxaR, perhaps in a FtsK-dependent manner. It is also intriguing that FtsK, an early divisome protein, appears to recruit CxaR to the septum only at later stages of septum synthesis. FtsK is a DNA translocase that promotes chromosome segregation into the two future daughter cells [22,44]. One could speculate that during late stages of septum synthesis, FtsK ceases its interaction with DNA and undergoes a conformational change that would be required for recruitment of CxaR to the septum, where it would promote degradation of Sle1.

The multiple levels of regulation of one of the 18 autolysins present in *S. aureus* highlight the critical importance of tightly controlling this class of potentially lethal enzymes.

## Methods

### Bacterial strains and growth conditions

The bacterial strains used in this work are listed in Table B in S1 Text. *S. aureus* was cultivated in tryptic soy broth (TSB, Difco) or on tryptic soy agar plates (TSA, VWR) at either 37°C or 30°C under aerated conditions. *Escherichia coli* strains were grown in Luria–Bertani (LB) broth (VWR) with shaking or on LB agar (VWR), at 37°C or 30°C. Where necessary, growth media were supplemented with 5-bromo-4-chloro-3-indolyl β-D-galactopyranoside (X-gal, 100µg/ml, Apollo Scientific), CdCl₂ (0.1µM-1µM), BCIP (5-bromo-4-chloro-3-indolyl phosphate p-toluidine, 100μg/ml, Sigma Aldrich), ampicillin (100µg/ml, Apollo Scientific), erythromycin (10µg/ml, Apollo Scientific), and/or combinations of kanamycin and neomycin (25 or 50µg/ml each, Apollo Scientific).

### Construction of plasmids and bacterial strains

The plasmids constructed and used in this study are described in Table C in S1 Text and the primers in Table D in S1 Text.

To delete or inactivate *cxaR*, two plasmids were constructed: pMAD-∆*cxaR,* designed to delete the entire *cxaR* gene from the *S. aureus* genome, and pMAD-CxaR^STOP^, engineered to introduce premature stop codon after *cxaR’*s ninth codon. pMAD-∆*cxaR* was constructed by amplifying the regions upstream (693 bp) and downstream (778 bp) of the *cxaR* gene from the *S. aureus* JE2 genome using the primer pairs KO_0710_P1_BamHI/KO_0710_P2 and KO_0710_P3/KO_0710_P4_SmaI, respectively. For the construction of pMAD-CxaR^STOP^ two 720 bp regions flanking the site of the stop codon insertion (between the ninth and tenth codons of *cxaR*) were amplified from the JE2 genome using the primer pairs KO_0710_P1_BamHI/STOP_0710_P2 and STOP_0710_P3/KO_0710_P4_SmaI. Primers STOP_0710_P2 and STOP_0710_P3 were designed to introduce the premature stop codon.

In both cases, the corresponding fragment pairs were fused by overlap extension PCR using primers KO_0710_P1_BamHI and KO_0710_P4_SmaI. The final PCR products were digested with BamHI and SmaI, cloned into the pMAD vector, and verified by sequencing. Both plasmids were electroporated into RN4220 (selection with erythromycin, 30°C) and subsequently transduced, using phage 80α, to COLΔ*phoB* Sle1-PhoB ∆*ftsK* strain, using previously described methodologies [45,46]. Additionally, pMAD-∆*cxaR* was transduced into JE2, NCTC8325-4, 8325-4Δ*sle1,* 8325-4 ClpX^R95C^, 8325-4 ClpX-FLAG and COLΔ*phoB* Sle1-PhoB strains. Deletion of *cxaR* or introduction of the premature stop codon was achieved through a two-step homologous recombination process. In the first step, chromosomal integration of the plasmid was selected at the non-permissive temperature of 43°C. In the second step, excision of the integrated plasmid, and thus loss of the *lacZ* and *erm* genes, was obtained after growth at the permissive temperature (30°C) without antibiotic selection. Successful deletion or mutation of *cxaR*, resulting in the generation of strains COLΔ*phoB* Sle1-PhoB ∆*ftsK* ∆*cxaR,* COLΔ*phoB* Sle1-PhoB ∆*ftsK* CxaR^V9X^, JE2∆*cxaR*, 8325-4∆*cxaR*, 8325-4Δ*sle1*∆*cxaR,* 8325-4 ClpX^R95C^ ∆*cxaR,* 8325-4 ClpX-FLAG ∆*cxaR* and COLΔ*phoB* Sle1-PhoB ∆*cxaR,* was confirmed by PCR and sequencing.

The COLΔ*phoB* Sle1-PhoB ∆*ftsK* strain was generated by transducing plasmid pBCBHV012 [44] into COLΔ*phoB* Sle1-PhoB, followed by double-crossover allelic exchange as described above. Mutants in which the *ftsK* gene was successfully deleted were identified by PCR.

Plasmids pCNX-CxaR and pCNX-CxaR-GFP, designed for *in trans* expression of CxaR or its C-terminal GFP fusion under the control of the inducible P*cad* promoter, were constructed as follows: the *cxaR* gene, including its Shine-Dalgarno sequence, was amplified using either the primer pair 0710Cterm_P1_SalI/ 0710Nterm_P2_KpnI (to amplify the entire gene including the stop codon) or 0710Cterm_P1_SalI/ 0710Cterm_P2_SmaI (to amplify the gene without the stop codon, for fusion to *gfp*). The PCR products were digested with SalI/KpnI or SalI/SmaI, respectively, and ligated into the corresponding vectors: pCNX [12] for expression of CxaR alone, or pCNX-GFPc [22] for expression of the GFP fusion. All constructs were verified by sequencing and pCNX-CxaR was introduced into *S. aureus* RN4220 cells via electroporation, using kanamycin/neomycin for selection. Phage 80α transduction was employed to transfer pCNX-CxaR and pCNX [12] empty vector into 8325-4∆*cxaR,* generating respectively 8325-4∆*cxaR* pCNX-CxaR and 8325-4∆*cxaR* pCNX.

To generate *S. aureus* strains expressing a gene encoding the CxaR-GFP fusion protein as the sole copy in the cell, under the control of its native promoter and from the native *cxaR* locus, the plasmid pMAD-CxaR-GFP was constructed using NEBuilder HiFi DNA Assembly (New England Biolabs). For the assembly, plasmid pMAD was digested with SmaI and two DNA fragments were amplified: (i) a 1228 bp fragment containing the CxaR-GFP coding sequence, amplified from the above described pCNX-CxaR-GFP plasmid using primers 0710GFP_P1HiFi and 0710GFP_P2; and (ii) a 520 bp fragment corresponding to the downstream region of *cxaR*, amplified from the JE2 genome using primers 0710GFP_P3 and 0710GFP_P2HiFi. The linearized plasmid and the two PCR fragments were assembled using NEBuilder HiFi DNA Assembly Mix (New England Biolabs), at 50°C for 1 hour, according to the manufacturer’s instructions. The resulting construct, pMAD-CxaR-GFP, was verified by PCR and sequencing of the inserted fragments. The plasmid was then introduced into electrocompetent *S. aureus* RN4220 cells (30°C, with erythromycin selection) and phage 80α was used to transduce it into NCTC8325-4, 8325-4Δ*ftsK,* 8325-4Δ*clpX* and 8325-4 ClpX^R95C^. The exchange of *cxaR* for *cxaR-_sf_gfp* was obtained after an integration/excision process, as described above, and confirmed by PCR. The final strains were named 8325-4 CxaR-GFP, 8325-4Δ*ftsK* CxaR-GFP, 8325-4Δ*clpX* CxaR-GFP and 8325-4 ClpX^R95C^ CxaR-GFP.

To generate a *S. aureus* strain expressing a C-terminal FLAG-tagged ClpX (3×FLAG; DYKDDDDKDYKDDDDKDYKDDDDK) as the sole cellular ClpX protein, we constructed plasmid pMAD-ClpX-3XFLAG. First, *clpX* 898 bp downstream region was amplified using primers DOWN_ClpX_SalI and ClpX_P4_SmaI and cloned into pMAD between SalI and SmaI restriction sites. Next, a 978 bp fragment containing the 3′ end of *clpX* fused to 3XFLAG coding sequence was amplified with primers ClpXDNT_P1_BamHI and ClpX_3XFLAG_P2_SalI, restricted with BamHI and SalI and cloned into pMAD, upstream of the previously introduced fragment. All inserts were confirmed by sequencing. The resulting plasmid, pMAD-ClpX-3XFLAG, was electroporated into RN4220 at 30°C under erythromycin selection and subsequently transduced into the wild-type strain NCTC8325-4. Chromosomal integration of the FLAG tag at the *clpX* locus was achieved via the standard pMAD integration–excision procedure. Correct allelic replacement was verified by PCR and sequencing, and the final strain was designated 8325-4 ClpX-FLAG.

To generate an *S. aureus* strain producing a C-terminal TagRFP fusion to ClpX, we constructed plasmid pMAD-ClpX-TagRFP. Two DNA fragments were amplified by PCR for this purpose. The first fragment, comprising the final 931 bp of the *clpX* coding region (excluding the stop codon), was amplified from NCTC8325-4 genomic DNA using primers ClpXDNT_P1_BamHI and ClpXRFP_P2. The second fragment, containing a five–amino-acid linker followed by the TagRFP coding sequence, was amplified from vector pTag-RFP-C (Evrogen) using primers ClpXRFP_P3 and RFPrv2_SalI. These two fragments were fused by overlap PCR, and the resulting product was digested with BamHI and SalI before being cloned upstream of the previously described 898-bp *clpX* downstream region fragment in pMAD. The insert was sequence-verified, and the final construct was designated pMAD-ClpX-TagRFP. The pMAD-ClpX-TagRFP plasmid was introduced into *S. aureus* RN4220 and subsequently transduced into the wild-type strain NCTC8325-4. Chromosomal insertion of the TagRFP sequence downstream of *clpX* followed the pMAD integration–excision procedure and was confirmed by PCR. The resulting strain was named 8325-4 ClpX-TagRFP.

### Transposon mutant library preparation and screening for suppressor mutants of the *ftsK* deletion that restore Sle1 at *S. aureus* outer cell surface

A transposon mutant library was generated in the COLΔ*phoB* Sle1-PhoB Δ*ftsK* background, following a previously described methodology [47]. In preparation for this process, plasmids pTM378 and pTM381, encoding respectively an active and a truncated (control) transposase, were first introduced into COLΔ*phoB* Sle1-PhoB Δ*ftsK* via phage transduction. Selection was performed using kanamycin and neomycin, each at 25 μg/ml. The resulting strains, named COLΔ*phoB* Sle1-PhoB ∆*ftsK* pTM378 and COLΔ*phoB* Sle1-PhoB ∆*ftsK* pTM381 were cultured overnight at 30°C in liquid medium, then diluted and allowed to grow until reaching an OD₆₀₀ of 0.4 (∼10⁸ CFU/ml). Subsequently, 5 ml of each culture were harvested by centrifugation at 3000*g* for 5 minutes and resuspended in an equal volume of SGMM medium (10mM glucose, 2mM MgCl₂, 3.5mM CaCl₂, 0.1% casein hydrolysate, 0.5% NaCl, and 10mM MES buffer, pH 6.8). The cultures were then infected with a 1:1:1 mixture of three φ11 lysates, each carrying a transposon with a distinct promoter, at a multiplicity of infection (MOI) of 2. Infected cultures were incubated overnight at room temperature without agitation. The next day, cells were centrifuged again (3000*g*, 5 minutes), resuspended in 5 ml of TSB, and allowed to recover with mild shaking at 37°C for 2 hours. To identify suppressor mutants restoring Sle1 surface localization, the cultures were plated on TSA supplemented with erythromycin (5 μg/ml) for transposon selection and BCIP (5-bromo-4-chloro-3-indolyl phosphate p-toluidine, 100 μg/ml) as the PhoB reporter substrate. Plates were incubated for 48 hours at 37°C. Colonies exhibiting a blue color phenotype were selected as candidates, and transposon insertion sites were subsequently mapped.

### Proteome analysis

*S. aureus* strains JE2 and JE2Δ*cxaR* were cultured in TSB medium with shaking, in three independent biological replicates, until reaching an OD₆₀₀ of 0.65. From each culture, cells from a 50 ml aliquot were collected by centrifugation at 7200*g* for 10 minutes and resuspended in 400 µL of PBS supplemented with Complete Mini Protease Inhibitor Cocktail (Roche). Cells were mechanically lysed using glass beads and a MP FastPrep 24 homogenizer (2 cycles of 1 minute each). Glass beads and cell debris were removed through two consecutive centrifugation steps at 3400*g* for 1 minute each. Protein concentrations in the resulting whole-cell lysates were determined using the Bradford assay (BCA Protein Assay Kit, Pierce). Subsequently, samples (10 µg of total protein) were run on 12% Mini-PROTEAN TGX precast gels (Bio-Rad), were excised and subjected to mass spectrometry (MS) analysis at the Proteome Center Tübingen, Germany.

For in-gel digestion of proteins, gel pieces were destained by washing three times with 5 mM ammonium bicarbonate (ABC) in acetonitrile (ACN) (1:1, v/v) for 20 minutes. After a dehydration step with 100% ACN for 10 minutes, disulfide bonds were reduced with 10 mM dithiothreitol (DTT) in 20 mM ABC for 45 minutes at 56°C, and thiol groups of cysteine residues were prevented from reoxidation by carbamidomethylation with 55 mM iodoacetamide (IAA) in 20mM ABC for 45 minutes in the dark. Gel pieces were then washed two times with 5 mM ABC in ACN (1:1, v/v) for 20 minutes and dehydrated with 100% ACN for 15 minutes. After evaporation of the liquid in a vacuum centrifuge for 10 minutes, gel pieces were soaked in a solution of 12.5 ng/µl sequencing grade trypsin (Promega) in 20 mM ABC, pH 8.0 for 10 minutes at room temperature (RT), and then covered with 20 mM ABC. After in-gel digestion of proteins at 37°C overnight, peptides were extracted in three consecutive steps with different extraction buffers for 30 minutes: first 3% (v/v) trifluoroacetic acid (TFA) in 30% (v/v) ACN was added, followed by 0.5% (v/v) formic acid (FA) in 80% (v/v) ACN, and finally by 100% ACN. ACN was evaporated from pooled supernatants by vacuum centrifugation. All incubation steps during the digestion protocol were carried out under shaking.

For Liquid Chromatography with tandem mass spectrometry (LC MS/MS), peptides were desalted with C_18_ StageTips [48] and analysed on an EASY-nLC 1200 UHPLC coupled to a Q Exactive HF mass spectrometer (both Thermo Fisher Scientific) as described previously [49] with little modification: peptides were separated on the analytical column using a 46 minutes segmented gradient of 10-33-50% of HPLC solvent B (80% acetonitrile in 0.1% formic acid) at a flow rate of 200 nl/min.

In the mass spectrometer, MS and MS/MS spectra were generated at resolution 60k. Full MS target value and maximum IT were set to 3x10^6^ and 25 ms, respectively. In each scan cycle, the 7 most intense precursor ions were picked up. The MS/MS target value was set to 10^5^ charges with a maximum IT of 220 ms.

The MS data from all replicates were processed together using MaxQuant software suite v.2.2.0.0 [50]. Database search was performed using the Andromeda search engine [51], which is integrated in MaxQuant. MS/MS spectra were searched against a target-decoy *S. aureus* Uniprot database consisting of 2889 protein entries and 245 commonly observed contaminants. In database search, full specificity was required for trypsin. Up to two missed cleavages were allowed. Carbamidomethylation of cysteine was set as fixed modification, whereas oxidation of methionine and acetylation of protein N-terminus were set as variable modifications. Initial mass tolerance was set to 4.5 parts per million (ppm) for precursor ions and 0.5 dalton (Da) for fragment ions. Peptide, protein and modification site identifications were reported at a false discovery rate (FDR) of 0.01, estimated by the target/decoy approach [52]. Label-free algorithm was enabled, as was the “match between runs” option for samples within one biological replicate [53]. Label-free quantification (LFQ) protein intensities from the MaxQuant data output were used for relative protein quantification. Downstream bioinformatic analysis (two-sample t-tests and Vulcano plots) was performed using the Perseus software package, version 1.6.2.3. Data was filtered for contaminants, reverse and only identified by site entries. Two sample tests were performed, considering P < 0.05 to be statistically significant and setting SO=0. The mass spectrometry proteomics data have been deposited to the ProteomeXchange Consortium via the PRIDE partner repository with the dataset identifier PXD066791 and 10.6019/PXD066791.

### RNA extraction and quantitative polymerase chain reaction (qPCR)

Three biological replicates of *S. aureus* NCTC8325-4 wild-type strain and 8325-4∆*cxaR* knock-out mutant were grown in 100 ml TSB cultures at 37°C with shaking until an OD₆₀₀ of 0.65. At this point, an equal volume of TM stop buffer (10 mM Tris–HCl pH 7.2, 5 mM MgCl_2_, 25 mM sodium azide (NaN_3_) and 0.5 mg/ml chloramphenicol) was added to rapidly halt cellular processes. Cultures were then centrifuged at 4°C for 30 minutes at 4000 rpm and the resulting pellets were immediately frozen in liquid nitrogen and stored overnight at −80°C. Cell pellets were resuspended in 350 µL of RNase-free NR buffer from the NZY Total RNA Isolation Kit (cat. no. MB13402, NZYTech). Mechanical lysis was performed using acid-washed glass beads and a MP FastPrep-24 homogenizer (two cycles of 1 minute each). Following lysis, glass beads and cell debris were removed by two consecutive 1 minute centrifugation steps at 3400*g*. RNA was then extracted using the NZY Total RNA Isolation Kit according to the manufacturer’s instructions. The integrity of total RNA was assessed by electrophoresis on a 1.2% agarose gel, and RNA concentration was quantified using a NanoDrop OneC spectrophotometer (Thermo Fisher Scientific). To remove residual genomic DNA, 10 µg of total RNA from each sample was treated with the TURBO DNA-free Kit (cat. no. AM1907, Thermo Fisher Scientific) according to the manufacturer’s instructions. The absence of genomic DNA contamination was confirmed by performing control PCR reactions using the DNase-treated RNA as template.

cDNA synthesis was performed using 1 µg of DNase-treated RNA and the SensiFAST cDNA Synthesis Kit (cat. no. BIO-65054, Bioline), according to the manufacturer’s instructions. Reverse transcription followed by quantitative PCR (qPCR) was used to quantify *sle1* transcription levels. qPCR reactions were carried out on a Real-Time Thermal Cycler qTower^3^ G (Analytik Jena) using 5 ng of cDNA per reaction and the SensiFAST SYBR No-ROX Kit (Bioline), according to the supplier’s protocol. Efficiency of amplifications was verified by generating a standard curve with serial dilutions of cDNA. Relative quantification of gene expression was calculated using the 2^–ΔΔCt^ (Ct is cycle threshold) method [54] with *S. aureus gyrA* as the housekeeping reference gene. Primer pairs sle1FWDSet1/sle1REVSet1 for *sle1* and gyrAFWDSet1/gyrAREVSet1 for *gyrA* (Table D in S1 Text) were designed using the PrimeQuest tool (IDT). qPCR reactions were performed in technical triplicates for each of three biological replicates per sample. GraphPad Prism 6 (GraphPad Software) was used to perform the statistical analysis using two‐tailed Mann–Whitney *U* test. *P*‐values > 0.05 were considered not significant (ns).

### Western blot

*S. aureus* total cellular protein extracts were prepared for Sle1, ClpX-FLAG and CxaR-GFP detection by western blot. For this, 50 ml cultures were grown in TSB to an OD₆₀₀ of 0.65, harvested by centrifugation at 3000*g* for 15 minutes, and resuspended in 400 μL PBS. Cells were mechanically lysed using an MP FastPrep 24 homogenizer (2 cycles of 1 minute each). Glass beads and cellular debris were removed by two sequential centrifugations at 3400*g* for 1 minute, and the resulting supernatant was collected. Total protein concentration was determined using the Bradford assay with bovine serum albumin as standard (BCA Protein Assay Kit, Pierce). Equal amounts of total protein were resolved on 12% Mini-PROTEAN TGX precast gels (Bio-Rad) and transferred onto 0.2 µm nitrocellulose membranes using the Trans-Blot Turbo RTA Transfer Kit and the Trans-Blot Turbo system (Bio-Rad). For quantification of Sle1, ClpX-FLAG and CxaR-GFP bands, membranes were cut to separate the regions above and below ~55 KDa (for Sle1) or ~70KDa (for ClpX-FLAG and CxaR-GFP). The bottom part of the membrane was blocked with 5% non-fat milk and incubated overnight at 4°C with anti-Sle1 antibody (1:5000 dilution) [22], monoclonal anti‐FLAG M2 mouse antibody (Sigma Cat No. F1804; 1:2000 dilution) or rabbit polyclonal GFP antibody (Chromotek Cat No.50430‐2‐AP, 1:1000 dilution). Membranes were then incubated for 1h with Goat Anti‐Rabbit IgG StarBright Blue 700 Fluorescent Secondary Antibody (Bio‐Rad Cat No.12004161; 1:5000 dilution) or, for detection of ClpX-FLAG, with StarBright Blue 700 Goat Anti-Mouse IgG (Bio‐Rad Cat No. 12004158; 1:5000 dilution). An iBright Imaging System (Invitrogen) was used to detect the antibody signal. The top part of the membrane was used to label all high‐molecular weight proteins as a loading control. For that, membranes were incubated with Sypro‐Ruby stain (Invitrogen) according to the manufacturer’s instructions, and the dye signal was detected using the iBright Imaging system. Invitrogen iBright Analysis Software was used to quantify the intensities of Sle1, ClpX-FLAG and CxaR-GFP bands and total Sypro‐Ruby‐stained bands. The intensity of each Sle1, ClpX-FLAG or CxaR-GFP band in a total cell extract was normalized against the total amount of high‐molecular weight proteins in its own loaded extract. The determined signal for each sample was then normalized against the parental strain Sle1, ClpX-FLAG and CxaR-GFP signals in, respectively, NCTC8325‐4, 8325‐4 ClpX-FLAG and 8325‐4 CxaR-GFP protein extracts, present in the same membrane.

### Fluorescence microscopy

*S. aureus* strains were grown overnight at 37°C in TSB, supplemented, as needed, with kanamycin and neomycin (50 µg/ml each). The following day, cultures were diluted 1:500 into fresh TSB, supplemented, when necessary, with 1 µM cadmium chloride as an inducer, and incubated until reaching an OD₆₀₀ of 0.65.

When required, cells were labeled with the membrane dye Nile Red (5µg/ml, Invitrogen) for 5 minutes or/and the fluorescent D-amino acid HADA that was added to a final concentration of 250 µM, and cells were incubated with agitation at 37°C for 30 minutes.

Cells were harvested by centrifugation at 10000 rpm for 1 minute, resuspended in 20 µL of PBS and 1µL of this suspension was mounted on a microscopy slide with a 1.2% agarose (TopVision, Thermo Fisher Scientific) in PBS pad. Structured Illumination Microscopy (SIM) imaging was performed using an Elyra PS.1 microscope (Zeiss) with a Plan-Apochromat 63×/1.4 oil DIC M27 objective. SIM images were acquired using five grid rotations, with 34 μm grating period for the 561 nm laser (100 mW), 28 μm period for 488 nm laser (100 mW) and 23 μm period for 405 nm laser (50 mW). Images were captured using a Pco.edge 5.5 camera and reconstructed using ZEN software (black edition, 2012, version 8.1.0.484) based on a structured illumination algorithm, using synthetic, channel specific optical transfer functions and noise filter settings ranging from −6 to −8. For each strain, a minimum of three biological replicates was imaged.

### Time-lapse microscopy

Overnight cultures of 8325-4 CxaR-GFP strain were diluted 1:200 in TSB and grown at 37°C with agitation until they reached mid exponential phase (OD_600_ 0.4). Before imaging, fluorescent membrane-labelling dye FM4-64 (5 µg/ml) was added to 1ml of the exponential growth culture, which was then incubated for 20 minutes at 37°C with agitation. The culture was centrifuged at 10000*g* for 1 minute and the pellet resuspended in 50 µL of M9 minimal media (KH_2_PO_4_ 3.4 g/L, VWR; K_2_HPO_4_ 2.9 g/L, VWR; di-ammonium citrate 0.7 g/L, Sigma-Aldrich; sodium acetate 0.26 g/L, Merck; glucose 1% (w/v), Merck; MgSO_4_ 0.7 mg/L, Sigma-Aldrich; CaCl_2_ 7 mg/L, Sigma-Aldrich; casamino acids 1% (w/v), Difco; minimum essential medium amino acids 1×, Thermo Fisher Scientific; and minimum essential medium vitamins 1×, Thermo Fisher Scientific) and 1 µL of the suspension was mounted on a pad of 1.2% Topvision Agarose (Thermo Fisher Scientific) in M9 minimal media. Cells were kept at 37°C during the microscopy and were imaged at 4 minutes intervals.

Imaging was performed in a DeltaVision OMX SR microscope equipped with a hardware-based focus stability (HW UltimateFocus) and an environmental control module (set to 37°C). Z-stacks of three epifluorescence images with a step size of 500 nm were acquired using a 488 nm laser (100 mW, at 15% maximal power) for the GFP fusions, and a 568 nm laser (100 mW, at 60% maximal power) for the FM4-64 membrane dye, each with an exposure time of 50 ms. Fluorescence channel alignment was performed using SoftWoRx v7.2.1. and images were further processed using Fiji [55], also employed to generate representative crops.

### Analysis of *S. aureus* growth

To assess bacterial growth, overnight cultures of *S. aureus* strains JE2 and JE2Δ*cxaR* were diluted 1:500 into fresh TSB medium. Cultures were incubated at 37°C with shaking, and optical density at 600 nm was measured at 1-hour intervals over a 13-hour period.

### Bacterial two hybrid assay

For bacterial adenylate cyclase two-hybrid (BACTH) interaction studies, the genes *cxaR*, *sle1*, and a truncated version of *sle1* lacking the signal peptide coding region (*sle1*_*–SP*_) were amplified from the *S. aureus* NCTC8325-4 genome. Primer pairs used were: Sle1_FW_XmaI/Sle1_BTH4_SacI for *sle1*, SP_Sle1_BTH3_XmaI/Sle1_BTH4_SacI for *sle1*_*–SP*_, and CxaR_BTH_P1/CxaR_BTH_P2 for *cxaR*. Amplified *sle1* and *sle1*_*–SP*_ products were digested with SmaI and SacI and ligated into the BACTH vector pKNT25 [30] giving rise to pSle1T25 and pSle1-SPT25 respectively. The *cxaR* fragment was cloned into pUT18, pre-digested with the blunt-end restriction enzyme SmaI, using NEBuilder HiFi DNA Assembly (New England Biolabs), following the manufacturer’s instructions, to produce pCxaRT18. The inserts integrity was confirmed by sequencing.

The constructed plasmids and pClpXT25 [22] were co-transformed in different combinations into the *E. coli* BTH101 reporter strain (cya-deficient) [30], along with the appropriate control plasmids: pKT25-ZIP/pUT18C-ZIP (positive control) and pKNT25/pUT18 (negative control). Transformants were plated on LB agar supplemented with 40 µg/ml X-Gal, 0.5 mM IPTG, 100 µg/ml ampicillin, and 50 µg/m kanamycin, and incubated at 30°C. Individual colonies were resuspended in 10 µL of LB, and 3µL was spotted onto MacConkey agar containing 1% maltose, 0.5 mM IPTG, 100 µg/ml ampicillin, and 50 µg/ml kanamycin. Plates were incubated for 48 h at 30°C. Experiments were performed in triplicate.

### Search for CxaR homologs and maximum likelihood tree construction

Using CxaR sequence we did an initial search for homologs using BLAST [31] and found hits among various species of the *Staphylococcus* genera and close relatives, but also among other Firmicutes and Fusobacteria. We selected 65 sequences of potential homologs, which were aligned using the L-INS-i algorithm of MAFFT (version 7.526) [56] and the subsequent alignment was used to make a Hidden Markov Model (HMM) using the HMMER software (version 3.4) [57]. The HMM model was then uploaded to the HMMER webserver [58] to search for proteins from the Uniprot database (updated 01/2025) [32] that would fit in the model, and 348 sequences of potential homolog proteins were retrieved. To reduce the redundancy of this dataset, we used CD-HIT [59] to select representative sequences, with less than 90% of identity among them, leaving 179 sequences. The CxaR sequence was reintroduced at this point, making a total of 180 sequences in the dataset. The sequences were then aligned using the L-INS-i algorithm of MAFFT, the alignment was trimmed using TrimAL (version 1.5.0) [60] and the trimmed alignment was used to make a maximum likelihood tree with IQ-Tree (version 3.0.1) [61] using the LG + F + R5 model (selected by IQ-Tree model finder). Branch support was assessed by the UFBoot2 method [62] implemented in IQ-Tree. The tree was visualized and annotated using iTOL [63]. The relevant information about the sequences (length, automated classification and taxonomy of the organisms) was retrieved from Uniprot, and domain search was done with ScanProsite [64] in the Prosite webserver (06/2025).

## Supporting information

S1 Text **Table A:** List of proteins significantly upregulated or downregulated (≥3-fold change) in the *Staphylococcus aureus* JE2Δ*cxaR* mutant compared to the wild-type JE2 strain. Quantitative proteomics data obtained from three biological replicates. **Table B.** Bacterial strains used in this study. **Table C.** Plasmids used and constructed in this study. **Table D.** Primers used in this study. **Fig A.** Set up of screening for Sle1 negative regulators. (A) Top, COLΔ*phoB* Sle1-PhoB strain, a construct encoding a C-terminal fusion of Sle1 to PhoB, expresses and exports a chimeric Sle1-PhoB protein through the Sec pathway. Through PhoB activity, this chimeric protein converts 5-Bromo-4-chloro-3-indolyl phosphate (BCIP) into a blue colored product, resulting in blue colonies. Bottom, If the expression or export of Sle1-PhoB is impaired, or its proteolysis is enhanced, BCIP is not degraded, and colonies remain white. (B) COLΔ*phoB* Sle1-PhoB strain produces blue colonies in the presence of BCIP. Deletion of *ftsK* in this background results in proteolysis of Sle1-PhoB, and consequently in its disappearance from the cells surface, resulting in white colonies. A transposon insertion in the COLΔ*phoB* Sle1-PhoB Δ*ftsK* strain disrupting a negative regulator of Sle1 could allow Sle1-PhoB to bypass the need for FtsK, and its resulting expression and export would result in blue colonies. **Fig B.** Loss of CxaR increases Sle1 total cell levels and impairs cell integrity and growth, leading to lysis and premature septum splitting. (A) Graph showing quantification of total Sle1 levels by Western blot, using an anti-Sle1 antibody, of total protein extracts of wild-type JE2 and CxaR knock-out mutant JE2Δ*cxaR*. A representative blot is shown below, including the Sypro Ruby staining used for sample normalization; the arrow indicates the Sle1 band. An approximately 10-fold increase in Sle1 levels was observed upon deletion on *cxaR*. The graph shows the ratio of Sle1 levels in JE2Δ*cxaR* relative to the parental strain JE2 (dashed line). Data are presented as a bar with scatter plot with mean ± SD (n = 4 biological replicates). (B) Growth curves of *S. aureus* JE2 and JE2Δ*cxaR* strains in TSB at 37°C. The JE2Δ*cxaR* culture exhibits an initial increase in optical density up to OD₆₀₀ ≈ 0.5, followed by a decline, indicating lysis, and subsequent recovery. (C) Structured Illumination Microscopy (SIM) images of the JE2Δ*cxaR* mutant labelled with the membrane dye Nile Red (upper panel), the cell wall-incorporated fluorescent D-amino acid HADA (middle panel) and the overlay of the two channels (lower panel; cell wall signal in cyan, membrane signal in magenta). The phenotypes present in a ∆*cxaR* mutant are indicated: (i) lysed cells which did not incorporate HADA, (ii) lysed cells labelled with both dyes, and (a) cells where septum splitting occurs prematurely, before septum synthesis is completed, some of which are lysed. Representative image from 3 biological replicates. Scale bar 1 µm. **Fig C.** ClpX protein levels are not altered in the absence of CxaR. Quantification of total ClpX-FLAG levels in 8325-4 ClpX-FLAG ∆*cxaR* strain, by Western blot. The graph shows the ratio of ClpX-FLAG levels in 8325-4 ClpX-FLAG ∆*cxaR* relative to the parental strain 8325-4 ClpX-FLAG (dashed line). Data are presented as a bar with scatter plot with mean ± SD (n = 3 biological replicates). **Fig D.** Absence of CxaR leads to premature septum splitting and cell lysis. Individual SIM channels of the indicated *S. aureus* strains corresponding to Fig 2B–F. Cells were labelled with membrane dye Nile Red (left panel and magenta in the overlay right panel) and with fluorescent D-amino acid HADA (middle panel and cyan in the overlay right panel). Strains 8325-4Δ*cxaR* pCNX and 8325-4Δ*cxaR* pCNX-CxaR were grown in the presence of 1 µM cadmium chloride. Representative images from at least 3 biological replicates. Scale bars1 µm. **Fig E.** CxaR–GFP fusion may be hyperfunctional, resulting in low Sle1 levels. Quantification of total Sle1 levels by Western blot, using an anti-Sle1 antibody, of total protein extracts of indicated strains. A representative blot is shown below, including the Sypro Ruby staining used for sample normalization; the arrow indicates the Sle1 band. The graph shows the ratio of Sle1 levels in each strain relative to the wild-type strain NCTC8325-4 (dashed line). Data are presented as bar with scatter plot with mean ± SD (n = 3 biological replicates). **Fig F.** CxaR localizes in small rings/foci during part of the cell cycle in *S. aureus* cells. Time lapse microscopy of strain 8325-4 CxaR-GFP, labelled with membrane-labelling dye FM4-64 (magenta). CxaR-GFP signal (cyan) is shown as the maximum intensity projection of 3 planes acquired as a Z-stack. Images were acquired every 4 minutes. Over the course of the time-lapse, localized CxaR-GFP can be observed in every cell in the image. **Fig G.** CxaR-GFP protein levels are not altered in the absence of FtsK or ClpX. Quantification of total CxaR-GFP levels by Western blot of total protein extracts of indicated strains. A representative blot is shown below, including the Sypro Ruby staining used for sample normalization. The graph shows the ratio of CxaR-GFP levels in the FtsK and ClpX mutants relative to the parental strain 8325-4 CxaR-GFP expressing CxaR-GFP as the only CxaR copy in the cell (dashed line). Data are presented as bar with scatter plot with mean ± SD (n = 3 biological replicates). Arrow indicates CxaR-GFP band. **Fig H.** Interaction between ClpX and CxaR detected by bacterial two-hybrid assay. *E. coli* BTH101 cells co-transformed with the plasmid pairs indicated were spotted onto MacConkey agar supplemented with maltose. A pink/red coloration indicates interaction. **Fig I.** ClpX-TagRFP primarily localizes in foci near the septum. Structured Illumination Microscopy (SIM) images of the *S. aureus* 8325-4 ClpX-TagRFP strain, in which ClpX-TagRFP is the sole cellular copy expressed from the native *clpX* locus. The top panel shows ClpX-TagRFP fluorescence, the middle panel shows cell wall labeling with the fluorescent D-amino acid HADA, and the bottom panel presents the merged channels (cell wall in cyan, ClpX-TagRFP in magenta). Scale bar 1 µm.
(DOCX)
