# Supplementary material for: A new regulator of the Staphylococcus aureus peptidoglycan hydrolase Sle1
Source: PLoS Genet. 2025 Dec 31;21(12):e1011990. doi: 10.1371/journal.pgen.1011990 (PMC12755775; doi:10.1371/journal.pgen.1011990)
Supplement: S1 Text — Table A: List of proteins significantly upregulated or downregulated (≥3-fold change) in the Staphylococcus aureus JE2ΔcxaR mutant compared to the wild-type JE2 strain. Quantitative proteomics data obtained from three biological replicates. Table B. Bacterial strains used in this study. Table C. Plasmids used and constructed in this study. Table D. Primers used in this study. Fig A. Set up of screening for Sle1 negative regulators. (A) Top, COLΔphoB Sle1-PhoB strain, a construct encoding a C-terminal fusion of Sle1 to PhoB, expresses and exports a chimeric Sle1-PhoB protein through the Sec pathway. Through PhoB activity, this chimeric protein converts 5-Bromo-4-chloro-3-indolyl phosphate (BCIP) into a blue colored product, resulting in blue colonies. Bottom, If the expression or export of Sle1-PhoB is impaired, or its proteolysis is enhanced, BCIP is not degraded, and colonies remain white. (B) COLΔphoB Sle1-PhoB strain produces blue colonies in the presence of BCIP. Deletion of ftsK in this background results in proteolysis of Sle1-PhoB, and consequently in its disappearance from the cells surface, resulting in white colonies. A transposon insertion in the COLΔphoB Sle1-PhoB ΔftsK strain disrupting a negative regulator of Sle1 could allow Sle1-PhoB to bypass the need for FtsK, and its resulting expression and export would result in blue colonies. Fig B. Loss of CxaR increases Sle1 total cell levels and impairs cell integrity and growth, leading to lysis and premature septum splitting. (A) Graph showing quantification of total Sle1 levels by Western blot, using an anti-Sle1 antibody, of total protein extracts of wild-type JE2 and CxaR knock-out mutant JE2ΔcxaR. A representative blot is shown below, including the Sypro Ruby staining used for sample normalization; the arrow indicates the Sle1 band. An approximately 10-fold increase in Sle1 levels was observed upon deletion on cxaR. The graph shows the ratio of Sle1 levels in JE2ΔcxaR relative to the parental strain JE [file pgen.1011990.s001.docx]

**S1 Text**

**A new regulator of the *Staphylococcus aureus* peptidoglycan hydrolase Sle1**

Helena Veiga^1,*^, Adrian Izquierdo-Martinez^1^, Leonor B. Marques^1^, Mariana G. Pinho^1,*^

^1^Instituto de Tecnologia Química e Biológica António Xavier, Universidade Nova de Lisboa; Oeiras, Portugal.

**Table of contents**

| Table A – Quantitative proteomics data |
| --- |
| Table B – Strains used in this study. |
| Table C – Plasmids used and constructed in this study |
| Table D – Primers used in this study. |
| Figure A |
| Figure B |
| Figure C |
| Figure D |
| Figure E |
| Figure F |
| Figure G |
| Figure H |
| Figure I |

**Table A.** List of proteins significantly upregulated or downregulated (≥3-fold change) in the *Staphylococcus aureus* JE2Δ*cxaR* mutant compared to the wild-type JE2 strain. Quantitative proteomics data obtained from three biological replicates.

| **Uniprot ID** | **JE2 locus tag** | **Gene** | **Fold Change** | **p-value** |
| --- | --- | --- | --- | --- |
| Upregulated |  |  |  |  |
| Q2FIE1 | SAUSA300_0837 | *dltC* | 20.8 | 4.72E-02 |
| Q2FE79 | SAUSA300_2364 | *sbi* | 20.5 | 1.74E-05 |
| A0A0H2XFJ8 | SAUSA300_1092 | *pyrP* | 13.9 | 5.23E-04 |
| Q2FF25 | SAUSA300_2057 | *atpC* | 12.6 | 1.20E-03 |
| A0A0H2XFE1 | SAUSA300_1692 | *-* | 1.,9 | 5.11E-03 |
| A0A0H2XHH4 | SAUSA300_0769 | *-* | 9.9 | 4.32E-03 |
| **Q2FJH7** | **SAUSA300_0438** | ***sle1*** | **9.0** | **3.64E-03** |
| A0A0H2XF42 | SAUSA300_0986 | *cydA* | 8.2 | 6.07E-03 |
| Q2FHM8 | SAUSA300_1103 | *rpoZ* | 7.7 | 2.74E-03 |
| Q2FDM1 | SAUSA300_2573 | *isaB* | 7.6 | 4.77E-03 |
| Q2FHN4 | SAUSA300_1097 | *pyrF* | 7.5 | 2.72E-03 |
| A0A0H2XGW1 | SAUSA300_2265 | *-* | 7.0 | 1.24E-03 |
| A0A0H2XI88 | SAUSA300_0658 | *-* | 6.8 | 2.65E-04 |
| A0A0H2XHM4 | SAUSA300_2273 | *-* | 6.0 | 4.75E-04 |
| A0A0H2XHE2 | SAUSA300_0377 | *-* | 5.8 | 1.92E-04 |
| Q2FJ71 | SAUSA300_0554 | *nagB* | 5.7 | 6.02E-05 |
| Q2FEZ4 | SAUSA300_2088 | *luxS* | 4.9 | 8.79E-04 |
| A0A0H2XH75 | SAUSA300_1098 | *pyrE* | 4.9 | 1.01E-03 |
| A0A0H2XJ57 | SAUSA300_0890 | *oppF* | 4.7 | 5.61E-04 |
| Q2FHN5 | SAUSA300_1096 | *carB* | 4.5 | 7.71E-04 |
| A0A0H2XH56 | SAUSA300_2389 | *norB* | 4.0 | 1.69E-03 |
| A0A0H2XHP8 | SAUSA300_1711 | *putA* | 3.9 | 5.40E-03 |
| A0A0H2XHH6 | SAUSA300_1004 | *-* | 3.8 | 1.24E-02 |
| A0A0H2XIA8 | SAUSA300_1094 | *pyrC* | 3.7 | 2.38E-06 |
| A0A0H2XHY5 | SAUSA300_2086 | *-* | 3.6 | 7.94E-03 |
| A0A0H2XIN1 | SAUSA300_1095 | *carA* | 3.5 | 1.39E-04 |
| A0A0H2XGJ5 | SAUSA300_1853 | *-* | 3.3 | 9.60E-03 |
| Q2FHN8 | SAUSA300_1093 | *pyrB* | 3.3 | 7.55E-05 |
| A0A0H2XHE4 | SAUSA300_1867 | *-* | 3.3 | 3.04E-03 |
| A0A0H2XGI4 | SAUSA300_2000 | *vga* | 3.1 | 9.12E-03 |
| A0A0H2XFD5 | SAUSA300_0093 | *-* | 3.1 | 1.25E-02 |
|  |  |  |  |  |
| Downregulated |  |  |  |  |
| Q2FGH2 | SAUSA300_1511 | *rpmG2* | 15.2 | 9.40E-04 |
| A0A0H2XEE7 | SAUSA300_2343 | *-* | 8.3 | 7.52E-04 |
| A0A0H2XJ34 | SAUSA300_0553 | *-* | 8.2 | 9.74E-06 |
| A0A0H2XGU3 | SAUSA300_1859 | *-* | 5.9 | 7.49E-03 |
| A0A0H2XJX3 | SAUSA300_0943 | *-* | 5.7 | 1.51E-04 |
| A0A0H2XHK2 | SAUSA300_2565 | *clfB* | 5.6 | 1.39E-02 |
| Q2FHV6 | SAUSA300_1024 | *coaD* | 5.1 | 1.63E-02 |
| Q2FK44 | SAUSA300_0220 | *pflB* | 4.6 | 3.23E-03 |
| A0A0H2XGZ2 | SAUSA300_1011 | *-* | 4.1 | 1.48E-02 |
| A0A0H2XHH5 | SAUSA300_1478 | *-* | 4.0 | 1.16E-02 |
| A0A0H2XF73 | SAUSA300_1112 | *-* | 3.9 | 1.10E-02 |
| Q2FI10 | SAUSA300_0970 | *purQ* | 3.8 | 3.90E-03 |
| A0A0H2XI67 | SAUSA300_1361 | *-* | 3.7 | 5.36E-03 |
| A0A0H2XJD3 | SAUSA300_1786 | *ecsA* | 3.4 | 2.34E-02 |
| A0A0H2XI62 | SAUSA300_1050 | *-* | 3.4 | 9.32E-04 |
| A0A0H2XHU0 | SAUSA300_2346 | *nirB* | 3.2 | 2.32E-02 |
| Q2FIR2 | SAUSA300_0713 | *queF* | 3.2 | 5.08E-03 |
| Q2FK29 | SAUSA300_0235 | *ldh1* | 3.1 | 7.32E-03 |
| A0A0H2XIG3 | SAUSA300_1537 | - | 3.0 | 2.19E-03 |

**Table B.** Bacterial strains used in this study.

| Strains | Description | Source or reference |
| --- | --- | --- |
| ***Escherichia coli*** |  |  |
| DC10B | Δ*dcm* in DH10B background; Dam methylation only | (1) |
| BTH101 | Reporter strain for BTH system; *cya* deficient | (2) |
| ***Staphylococcus aureus*** |  |  |
| NCTC8325-4 | MSSA strain | R. Novick |
| COL | HA-MRSA strain | (3) |
| JE2 | CA-MRSA strain | (4) |
| RN4220 | Restriction-deficient derivative of NCTC8325-4 | (5) |
| COLΔ*phoB* Sle1-PhoB | COL Δ*phoB* Δ*sle1*::*sle1-phoB_-SP_* | (6) |
| COLΔ*phoB* Sle1-PhoB ∆*ftsK* | COL Δ*phoB* Δ*sle1*::*sle1-phoB_-SP_* ∆*ftsK* | This study |
| COLΔ*phoB* Sle1-PhoB ∆*ftsK* pTM378 | COL Δ*phoB* Δ*sle1*::*sle1-phoB_-SP_* ∆*ftsK* pTM378; Kan^r^ | This study |
| COLΔ*phoB* Sle1-PhoB ∆*ftsK* pTM381 | COL Δ*phoB* Δ*sle1*::*sle1-phoB_-SP_* ∆*ftsK* pTM381; Kan^r^ | This study |
| COLΔ*phoB* Sle1-PhoB ∆*ftsK* ∆*cxaR* | COL Δ*phoB* Δ*sle1*::*sle1-phoB_-SP_* ∆*ftsK* ∆*cxaR* | This study |
| COLΔ*phoB* Sle1-PhoB ∆*ftsK* CxaR^V9X^ | COLΔ*phoB* Δ*sle1*::*sle1-phoB_-SP_* ∆*ftsK* Δ*cxaR::cxaR*^V9X^ *; cxaR* truncated by an introduced stop codon. | This study |
| COLΔ*phoB* Sle1-PhoB ∆*cxaR* | COL Δ*phoB* Δ*sle1*::*sle1-phoB_-SP_* ∆*cxaR* | This study |
| JE2∆*cxaR* | JE2 ∆*cxaR* | This study |
| 8325-4∆*cxaR* | NCTC8325-4 Δ*cxaR* | This study |
| 8325-4Δ*ftsK* | NCTC8325-4 Δ*ftsK* | (7) |
| 8325-4Δ*clpX* | NCTC8325-4 Δ*clpX* | (6) |
| 8325-4 ClpX^R95C^ | NCTC8325-4 Δ*clpX::clpX*^R95C^ | (6) |
| 8325-4 ClpX^R95C^ ∆*cxaR* | NCTC8325-4 Δ*clpX::clpX*^R95C^ Δ*cxaR* | This study |
| 8325-4Δ*sle1* | NCTC8325-4 Δ*sle1* | (8) |
| 8325-4Δ*sle1*∆*cxaR* | NCTC8325-4 Δ*sle1* Δ*cxaR* | This study |
| 8325-4∆*cxaR* pCNX | NCTC8325-4 Δ*cxaR* pCNX; Kan^r^ | This study |
| 8325-4∆*cxaR* pCNX-CxaR | NCTC8325-4 Δ*cxaR* pCNX-CxaR; Kan^r^ | This study |
| 8325-4 CxaR-GFP | NCTC8325-4 Δ*cxaR::cxaR-_sf_gfp* | This study |
| 8325-4Δ*ftsK* CxaR-GFP | NCTC8325-4 Δ*ftsK* Δ*cxaR::cxaR-_sf_gfp* | This study |
| 8325-4Δ*clpX* CxaR-GFP | NCTC8325-4 Δ*clpX* Δ*cxaR::cxaR-_sf_gfp* | This study |
| 8325-4 ClpX^R95C^ CxaR-GFP | NCTC8325-4 Δ*clpX::clpX*^R95C^ Δ*cxaR::cxaR-_sf_gfp* | This study |
| 8325-4 ClpX-FLAG | NCTC8325-4 Δ*clpX::clpX-3XFLAG* | This study |
| 8325-4 ClpX-FLAG ∆*cxaR* | NCTC8325-4 Δ*clpX::clpX-3XFLAG* Δ*cxaR* | This study |
| 8325-4 ClpX-TagRFP | NCTC8325-4 Δ*clpX::clpX-tagRFP* | This study |

Abbreviations: Kan^R^ – Kanamycin resistance

**Table C.** Plasmids used and constructed in this study.

| Plasmid | Description | Source or Reference |
| --- | --- | --- |
| pMAD | *E. coli – S. aureus* shuttle vector with a thermosensitive origin of replication for Gram-positive bacteria; Amp^r^, Ery^r^ | (9) |
| pBCBHV012 | pMAD containing up- and downstream regions of *ftsK;* Amp^r^ Ery^r^ | (7) |
| pMAD-∆*cxaR* | pMAD containing up- and downstream regions of *cxaR;* Amp^r^ Ery^r^ | This study |
| pMAD-CxaR^STOP^ | pMAD containing a stop codon, and flanking regions, for insertion between codons 9 and 10 of *cxaR;* Amp^r^ Ery^r^ | This study |
| pMAD-CxaR-GFP | pMAD containing *cxaR* 3’ end-*_sf_gfp*-*cxaR* downstream region; Amp^r^ Ery^r^ | This study |
| pMAD-ClpX-3XFLAG | pMAD containing *clpX* 3’ end-*3xFLAG*-*clpX* downstream region; Amp^r^ Ery^r^ | This study |
| pMAD-ClpX-TagRFP | pMAD containing *clpX* 3’ end-*tagRFP*-*clpX* downstream region; Amp^r^ Ery^r^ | This study |
| pCNX | *E. coli – S. aureus* replicative plasmid with cadmium-inducible promoter P*cad*; Amp^r^, Kan^r^ | (8) |
| pCNX-CxaR | pCNX containing *cxaR* under P*cad* promoter*;* Amp^r^, Kan^r^ | This study |
| pCNX-GFPc | pCNX containing a multiple cloning site upstream of the coding sequences for a 5 aa linker and _sf_GFP*;* Amp^r^, Kan^r^ | (6) |
| pCNX-CxaR-GFP | pCNX containing *cxaR-_sf_gfp;* Amp^r^, Kan^r^ | This study |
| pTM378 | Plasmid for HMAR1 C9 transposase expression; Kan^r^ | (10) |
| pTM381 | Plasmid for ΔHMAR1 C9 truncated transposase expression; Kan^r^ | (10) |
| pUT18 | BTH plasmid, C-terminal CyaA_T18_ fusion; Amp^r^ | (2) |
| pKNT25 | BTH plasmid, C-terminal CyaA_T25_ fusion; Kan^r^ | (2) |
| pUT18C-ZIP | BTH control plasmid; Amp^r^ | (2) |
| pKT25-ZIP | BTH control plasmid; Kan^r^ | (2) |
| pClpXT25 | pKNT25 containing *clpX*-*cyaA*_T25_ fusion; Kan^r^ | (6) |
| pSle1T25 | pKNT25 containing *sle1*-*cyaA*_T25_ fusion; Kan^r^ | This study |
| pSle1-SPT25 | pKNT25 containing *sle1_-SP_*-*cyaA*_T25_ fusion; Kan^r^ | This study |
| pCxaRT18 | pUT18 containing*cxaR*-*cyaA*_T18_ fusion; Amp^r^ | This study |

Abbreviations: Amp^R^ – ampicillin resistance; Ery^R^ – erythromycin resistance; Kan^R^ – Kanamycin resistance; CyaA_T25_ and CyaA_T18_ - *B. pertussis* adenylate cyclase fragments T25 and T18.

**Table D.** Primers used in this study.

| Primer Name | Primer Sequence (5’- 3’)* | |
| --- | --- | --- |
| KO_0710_P1_BamHI | TGAGGATCCGACAATGTATGATGTAGTAGCGCG | |
| KO_0710_P2 | GAAATGTAATTAGGCAATGTCCTCCTATTTCTCC | |
| KO_0710_P3 | GGAGGACATTGCCTAATTACATTTCAATTATATTAGC | |
| KO_0710_P4_SmaI | TACTGCCCGGGGCGCCTGCACCAGCTATCATGGTTAC | |
| STOP_0710_P2 | CTCGCCATTCAA**TTA**TACATGTTTTTTTATAAGGTGTTGC | |
| STOP_0710_P3 | CCTTATAAAAAAACATGTA**TAA**TTGAATGGCGAGTTTGATTTAGTACG |  |
| 0710Cterm_P1_SalI | GCTGCGCTGTCGACTAGGAGGACATTGCCATGCAAC | |
| 0710Nterm_P2_KpnI | GCTGCGGTACCTTAACGTGCATTACGTTGGTG | |
| 0710Cterm_P2_SmaI | TACTGCCCGGGACGTGCATTACGTTGGTGTAATTC | |
| 0710GFP_P1HiFi | CGATGCATGCCATGGTACCCGCAACACCTTATAAAAAAACATG | |
| 0710GFP_P2 | AATTGAAATGTAATTATTTGTATAGTTCATCCATGCC | |
| 0710GFP_P3 | CTATACAAATAATTACATTTCAATTATATTAGC | |
| 0710GFP_P2HiFi | GCTTCTAGAATTCGAGCTCCCCCTGCGCCCACTACAATGCCGAC | |
| Sle1_FW_XmaI | TACTCCCCGGGGCAAGAGGAGGATTTTAAAGTGC | |
| Sle1_BTH4_SacI | GCTGCGAGCTCCCGTGAATATATCTATAATTATTTAC | |
| SP_Sle1_BTH3_XmaI | TACTGCCCGGGGCAAGAGGAGGATTTTAAAGTGGCTACAACTCACACAGTAAAACCGGG | |
| CxaR_BTH_P1 | GGTCGACTCTAGAGGATCCCCTAGGAGGACATTGCCATGCAACACC | |
| CxaR_BTH_P2 | GAATTCGAGCTCGGTACCCGACGTGCATTACGTTGGTGTAATTC | |
| ClpXDNT_P1_BamHI | TGAGGATCCGGTAGTGGTAAAACATTATTAGC | |
| ClpX 3XFLAG_P2_SalI | GCTGCGTCGAC**TTA***TTTATCGTCATCATCCTTGTAGTCTTTATCGTCGTCATCTTTATAATCTTTGTCATCATCATCTTTGTAATC*AGCTGATGTTTTACTATTATTAATTAAATTGCC | |
| DOWN ClpX_SalI | GCTGCGTCGACTCATTGATGTGTTATAAAAGTG | |
| ClpX_P4_SmaI | TACTCCCCGGGGTACGATGCTTAAATAAATTATAATC | |
| ClpXRFP_P2 | CGCCCTTAGACACGGAGGCGCCGCAGGAAGCTGATGTTTTACTATTATTAA | |
| ClpXRFP_P3 | CATCAGCTTCCTGCGGCGCCTCCGTGTCTAAGGGCGAAGAGCTG | |
| RFPrv2_SalI | GCTGCGTCGACTTAATTAAGTTTGTGCCCCAG | |
| sle1FWDSet1 | CGTCGTGCTGAAATTGGTAAAG | |
| sle1REVSet1 | ACCTACATCTGTTTGAGCGATAG | |
| gyrAFWDSet1 | GCAGCAGCAATGCGTTATAC | |
| gyrAREVSet1 | ACTGACGGCTCTCTTTCATTAC | |

*Underlined sequences indicate restriction sites, italicized letters denote the 3×FLAG tag, and sequences in bold represent a stop codon introduced with the primers.


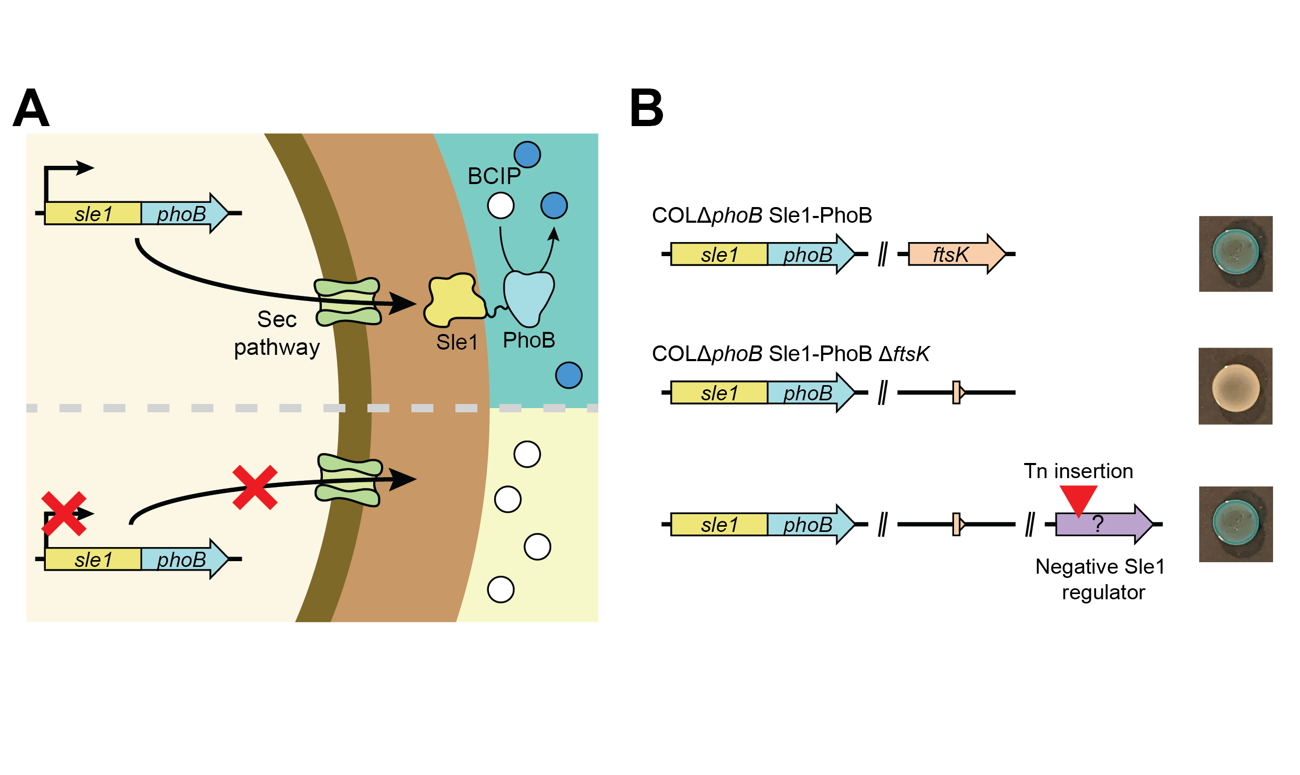


**Fig A.** **Set up of screening for Sle1 negative regulators.** **(A)** Top, COLΔ*phoB* Sle1-PhoB strain, a construct encoding a C-terminal fusion of Sle1 to PhoB, expresses and exports a chimeric Sle1-PhoB protein through the Sec pathway. Through PhoB activity, this chimeric protein converts 5-Bromo-4-chloro-3-indolyl phosphate (BCIP) into a blue colored product, resulting in blue colonies. Bottom, If the expression or export of Sle1-PhoB is impaired, or its proteolysis is enhanced, BCIP is not degraded, and colonies remain white. (**B)** COLΔ*phoB* Sle1-PhoB strain produces blue colonies in the presence of BCIP. Deletion of *ftsK* in this background results in proteolysis of Sle1-PhoB, and consequently in its disappearance from the cells surface, resulting in white colonies. A transposon insertion in the COLΔ*phoB* Sle1-PhoB Δ*ftsK* strain disrupting a negative regulator of Sle1 could allow Sle1-PhoB to bypass the need for FtsK, and its resulting expression and export would result in blue colonies.


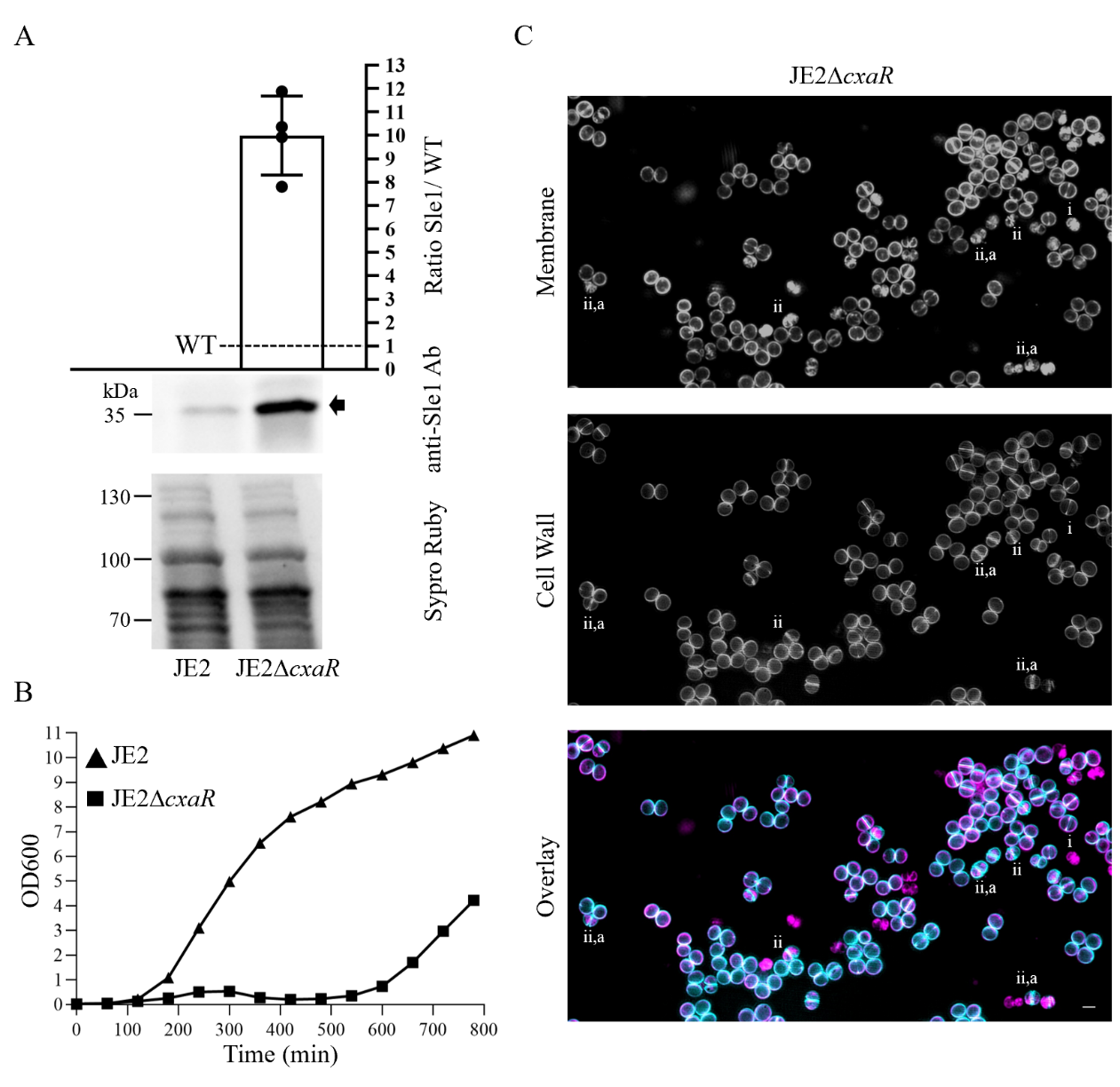
**Fig B. Loss of CxaR increases Sle1 total cell levels and impairs cell integrity and growth, leading to lysis and premature septum splitting. (A)** Graph showing quantification of total Sle1 levels by Western blot, using an anti-Sle1 antibody, of total protein extracts of wild-type JE2 and CxaR knock-out mutant JE2Δ*cxaR.* A representative blot is shown below, including the Sypro Ruby staining used for sample normalization; the arrow indicates the Sle1 band. An approximately 10-fold increase in Sle1 levels was observed upon deletion on *cxaR*. The graph shows the ratio of Sle1 levels in JE2Δ*cxaR* relative to the parental strain JE2 (dashed line). Data are presented as a bar with scatter plot with mean ± SD (n = 4 biological replicates). **(B)** Growth curves of *S. aureus* JE2 and JE2Δ*cxaR* strains in TSB at 37°C. The JE2Δ*cxaR* culture exhibits an initial increase in optical density up to OD₆₀₀ ≈ 0.5, followed by a decline, indicating lysis, and subsequent recovery. **(C)** Structured Illumination Microscopy (SIM) images of the JE2Δ*cxaR* mutant labelled with the membrane dye Nile Red (upper panel), the cell wall-incorporated fluorescent D-amino acid HADA (middle panel) and the overlay of the two channels (lower panel; cell wall signal in cyan, membrane signal in magenta). The phenotypes present in a ∆*cxaR* mutant are indicated: (i) lysed cells which did not incorporate HADA, (ii) lysed cells labelled with both dyes, and (a) cells where septum splitting occurs prematurely, before septum synthesis is completed, some of which are lysed. Representative image from 3 biological replicates. Scale bar 1 µm.

**
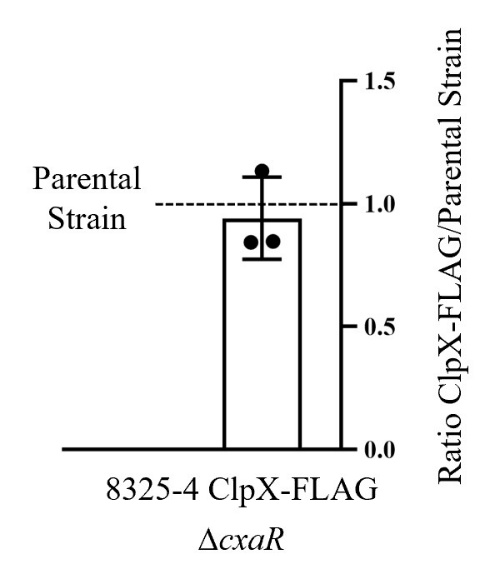
**

**Fig C. ClpX protein levels are not altered in the absence of CxaR.** Quantification of total ClpX-FLAG levels in 8325-4 ClpX-FLAG ∆*cxaR* strain, by Western blot. The graph shows the ratio of ClpX-FLAG levels in 8325-4 ClpX-FLAG ∆*cxaR* relative to the parental strain 8325-4 ClpX-FLAG (dashed line). Data are presented as a bar with scatter plot with mean ± SD (n = 3 biological replicates).


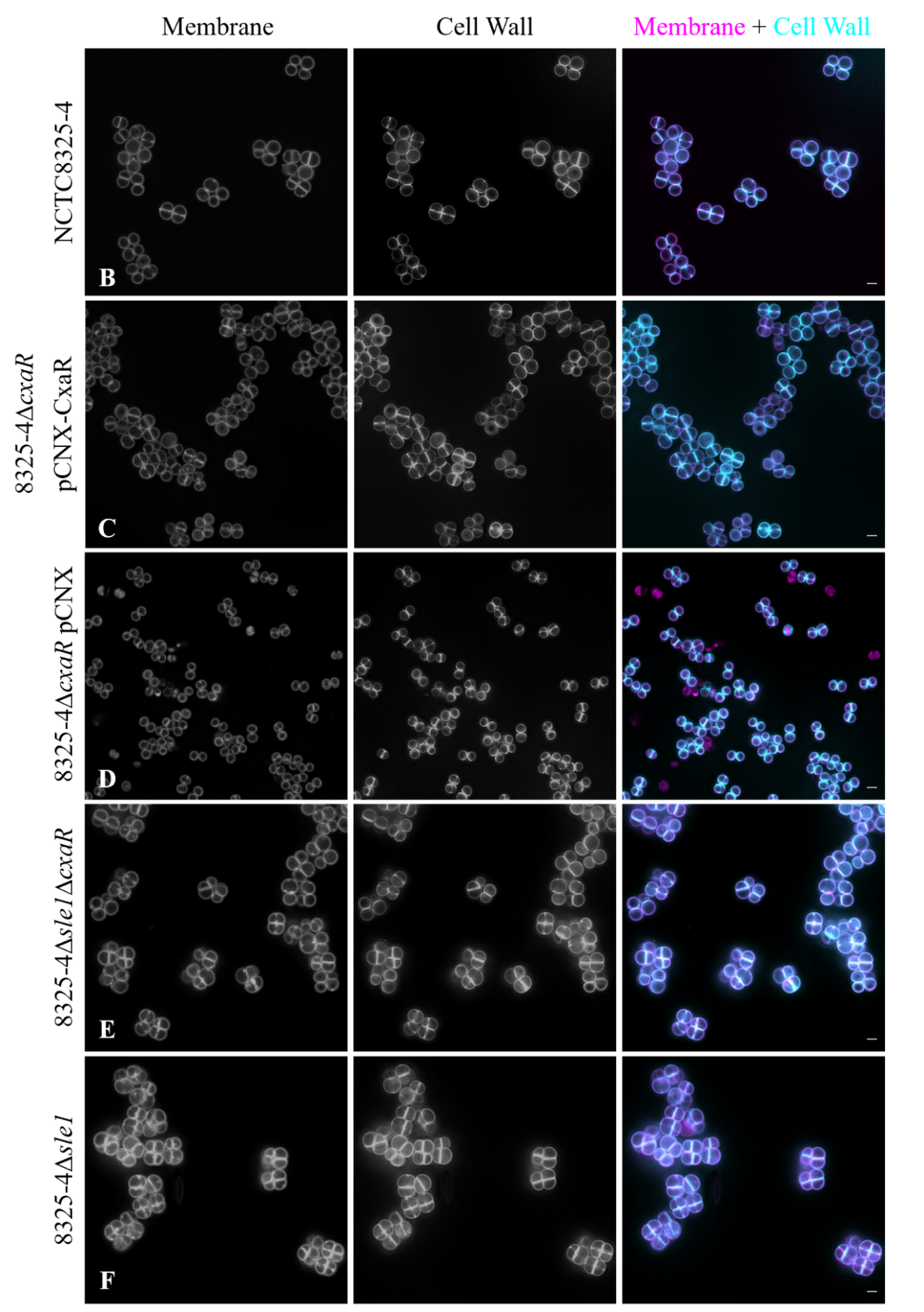
**Fig D. Absence of CxaR leads to premature septum splitting and cell lysis.** Individual SIM channels of the indicated *S. aureus* strains corresponding to figure 2B-F. Cells were labelled with membrane dye Nile Red (left panel and magenta in the overlay right panel) and with fluorescent D-amino acid HADA (middle panel and cyan in the overlay right panel). Strains 8325-4Δ*cxaR* pCNX and 8325-4Δ*cxaR* pCNX-CxaR were grown in the presence of 1 µM cadmium chloride. Representative images from at least 3 biological replicates. Scale bars1 µm.


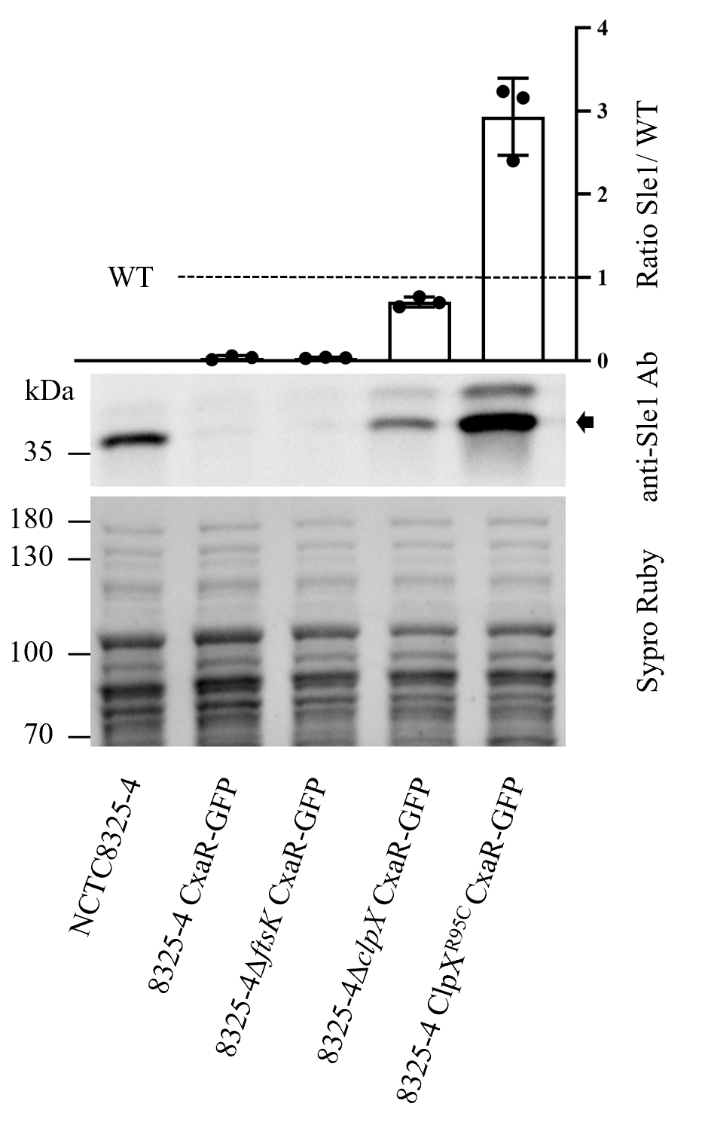


**Fig E. CxaR–GFP fusion may be hyperfunctional, resulting in low Sle1 levels.** Quantification of total Sle1 levels by Western blot, using an anti-Sle1 antibody, of total protein extracts of indicated strains. A representative blot is shown below, including the Sypro Ruby staining used for sample normalization; the arrow indicates the Sle1 band. The graph shows the ratio of Sle1 levels in each strain relative to the wild-type strain NCTC8325-4 (dashed line). Data are presented as bar with scatter plot with mean ± SD (n = 3 biological replicates).


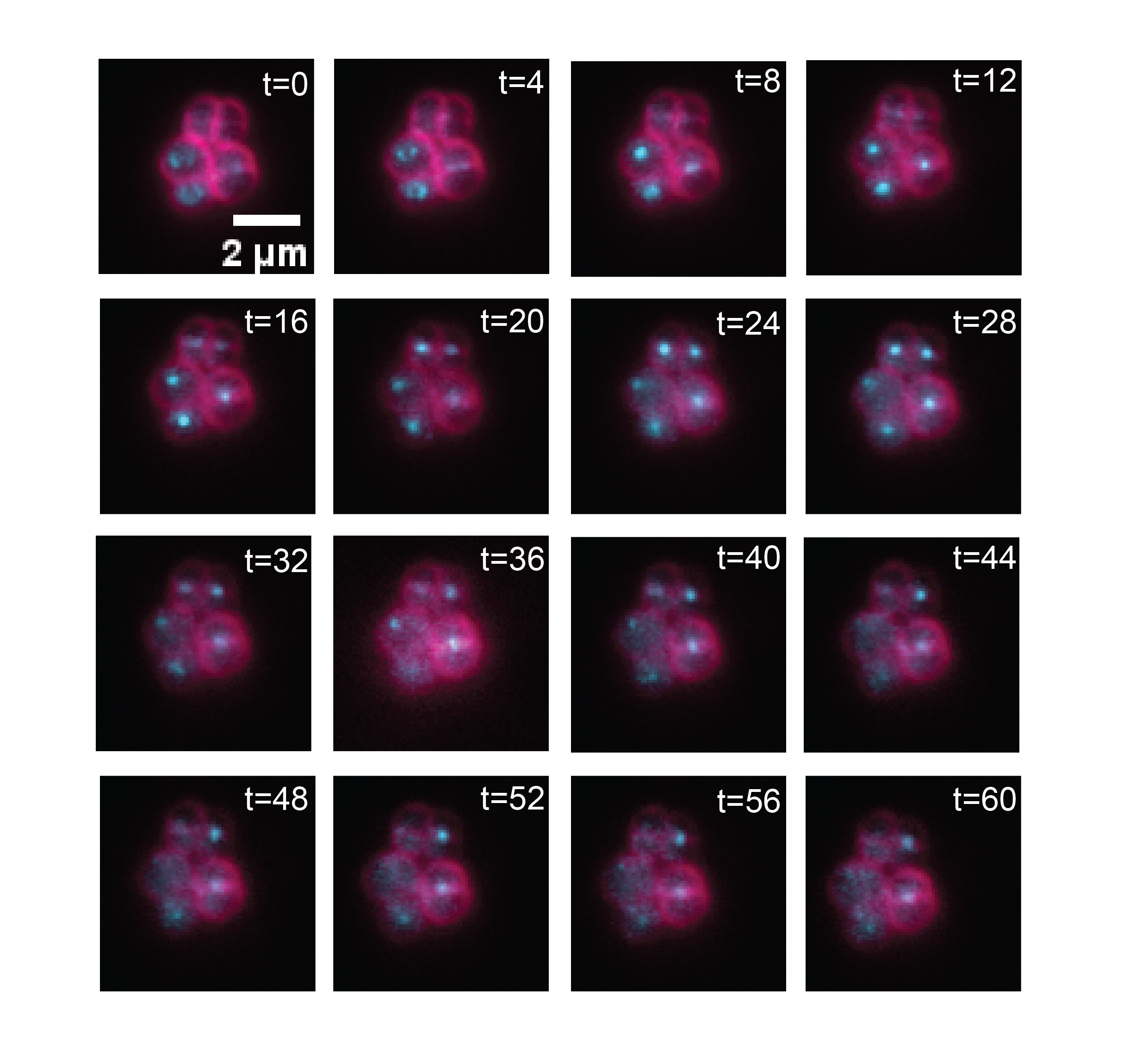


**Fig F. CxaR localizes in small rings/foci during part of the cell cycle in *S. aureus* cells.** Time lapse microscopy of strain 8325-4 CxaR-GFP, labelled with membrane-labelling dye FM4-64 (magenta). CxaR-GFP signal (cyan) is shown as the maximum intensity projection of 3 planes acquired as a Z-stack. Images were acquired every 4 minutes. Over the course of the time-lapse, localized CxaR-GFP can be observed in every cell in the image.

**
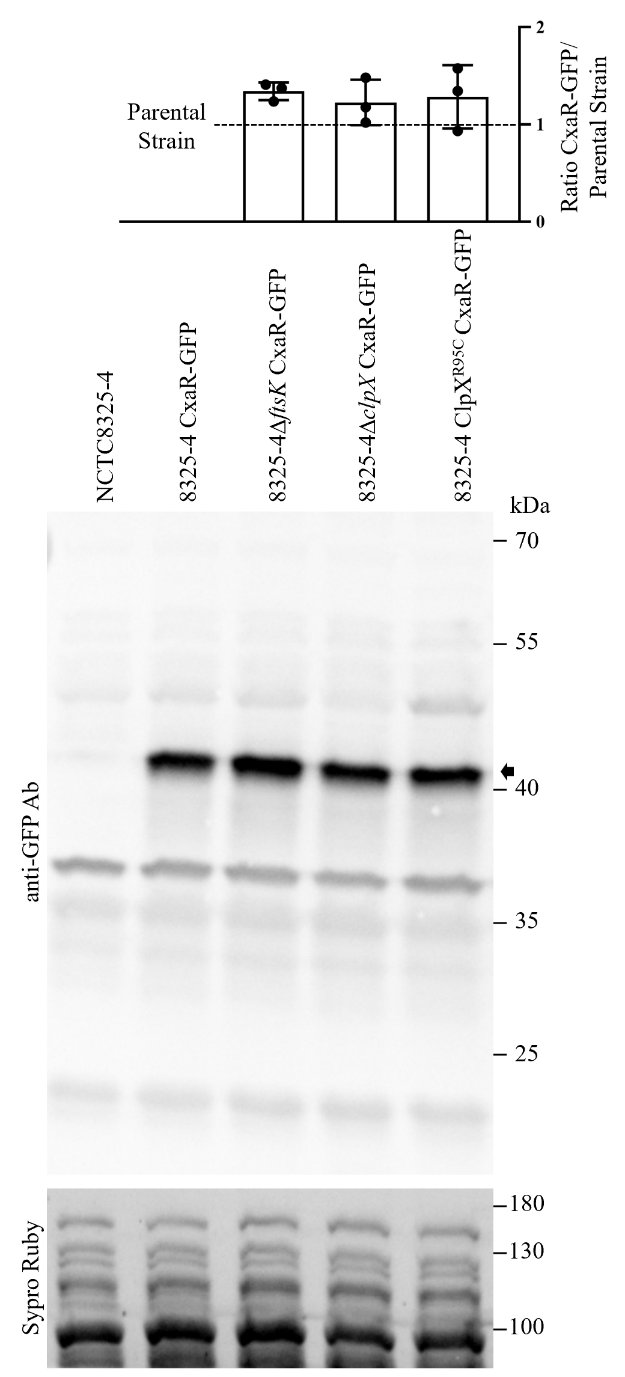
**

**Fig G. CxaR-GFP protein levels are not altered in the absence of FtsK or ClpX.** Quantification of total CxaR-GFP levels by Western blot of total protein extracts of indicated strains. A representative blot is shown below, including the Sypro Ruby staining used for sample normalization. The graph shows the ratio of CxaR-GFP levels in the FtsK and ClpX mutants relative to the parental strain 8325-4 CxaR-GFP expressing CxaR-GFP as the only CxaR copy in the cell (dashed line). Data are presented as bar with scatter plot with mean ± SD (n = 3 biological replicates). Arrow indicates CxaR-GFP band.

**
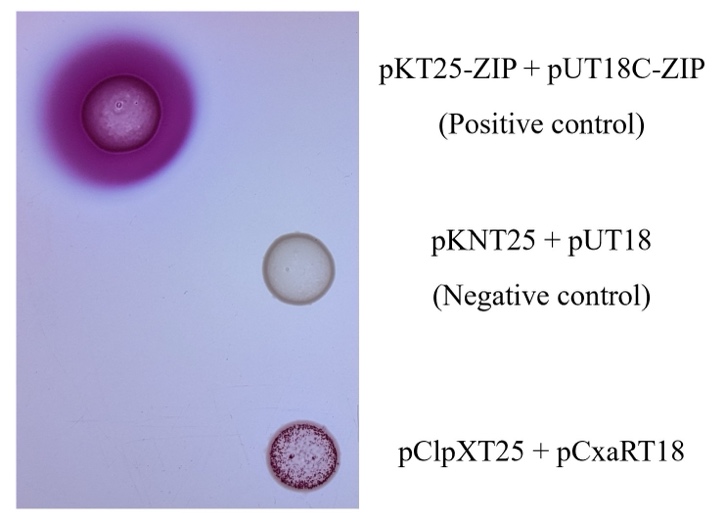
**

**Fig H. Interaction between ClpX and CxaR detected by bacterial two-hybrid assay.** *E. coli* BTH101 cells co-transformed with the plasmid pairs indicated were spotted onto MacConkey agar supplemented with maltose. A pink/red coloration indicates interaction.

**
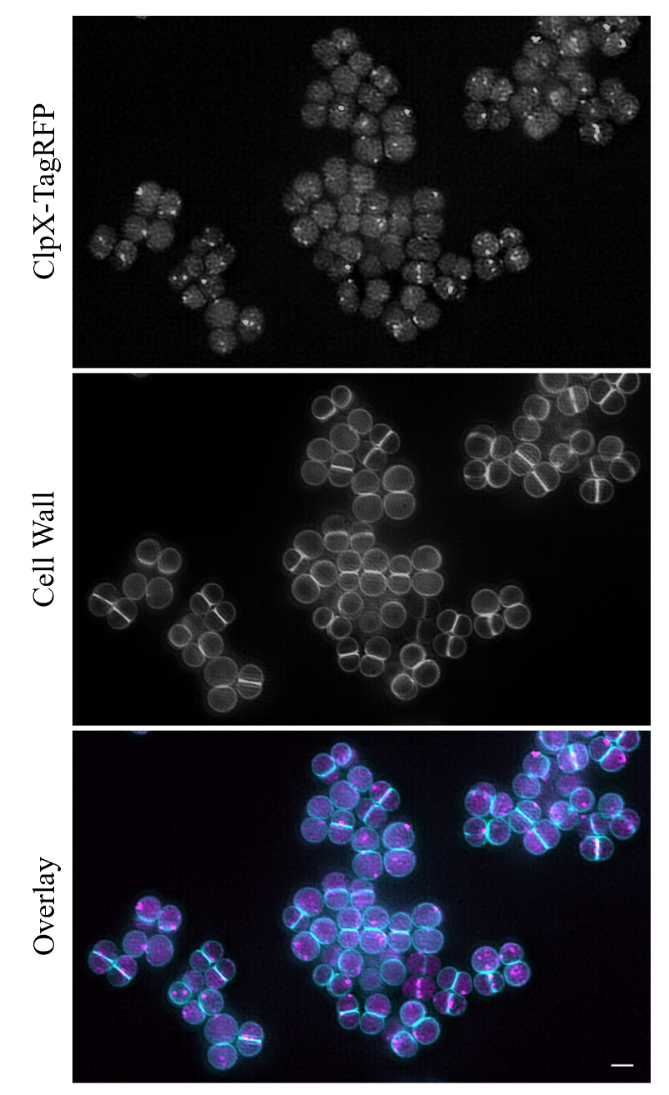
**

**Fig I. ClpX-TagRFP primarily localizes in foci near the septum.** Structured Illumination Microscopy (SIM) images of the *S. aureus* 8325-4 ClpX-TagRFP strain, in which ClpX-TagRFP is the sole cellular copy expressed from the native *clpX* locus. The top panel shows ClpX-TagRFP fluorescence, the middle panel shows cell wall labeling with the fluorescent D-amino acid HADA, and the bottom panel presents the merged channels (cell wall in cyan, ClpX-TagRFP in magenta). Scale bar 1 µm.

**Bibliography**

1. Monk IR, Shah IM, Xu M, Tan MW, Foster TJ. 2012. Transforming the untransformable: application of direct transformation to manipulate genetically *Staphylococcus aureus* and *Staphylococcus epidermidis*. mBio 3:e00277-11.

2. Karimova G, Pidoux J, Ullmann A, Ladant D. 1998. A bacterial two-hybrid system based on a reconstituted signal transduction pathway. Proc Natl Acad Sci U S A 95:5752-5756.

3. Gill SR, Fouts DE, Archer GL, Mongodin EF, Deboy RT, Ravel J, Paulsen IT, Kolonay JF, Brinkac L, Beanan M, Dodson RJ, Daugherty SC, Madupu R, Angiuoli SV, Durkin AS, Haft DH, Vamathevan J, Khouri H, Utterback T, Lee C, Dimitrov G, Jiang L, Qin H, Weidman J, Tran K, Kang K, Hance IR, Nelson KE, Fraser CM. 2005. Insights on evolution of virulence and resistance from the complete genome analysis of an early methicillin-resistant *Staphylococcus aureus* strain and a biofilm-producing methicillin-resistant *Staphylococcus epidermidis* strain. J Bacteriol 187:2426-2438.

4. Fey PD, Endres JL, Yajjala VK, Widhelm TJ, Boissy RJ, Bose JL, Bayles KW. 2013. A genetic resource for rapid and comprehensive phenotype screening of nonessential *Staphylococcus aureus* genes. mBio 4:e00537-12.

5. Nair D, Memmi G, Hernandez D, Bard J, Beaume M, Gill S, Francois P, Cheung AL. 2011. Whole-genome sequencing of *Staphylococcus aureus* strain RN4220, a key laboratory strain used in virulence research, identifies mutations that affect not only virulence factors but also the fitness of the strain. J Bacteriol 193:2332-2335.

6. Veiga H, Jousselin A, Schaper S, Saraiva BM, Marques LB, Reed P, Wilton J, Pereira PM, Filipe SR, Pinho MG. 2023. Cell division protein FtsK coordinates bacterial chromosome segregation and daughter cell separation in *Staphylococcus aureus*. EMBO J 42:e112140.

7. Veiga H, Pinho MG. 2017. *Staphylococcus aureus* requires at least one FtsK/SpoIIIE protein for correct chromosome segregation. Mol Microbiol 103:504-517.

8. Monteiro JM, Fernandes PB, Vaz F, Pereira AR, Tavares AC, Ferreira MT, Pereira PM, Veiga H, Kuru E, VanNieuwenhze MS, Brun YV, Filipe SR, Pinho MG. 2015. Cell shape dynamics during the staphylococcal cell cycle. Nat Commun 6:8055.

9. Arnaud M, Chastanet A, Debarbouille M. 2004. New vector for efficient allelic replacement in naturally nontransformable, low-GC-content, gram-positive bacteria. Appl Environ Microbiol 70:6887-6891.

10. Wang H, Claveau D, Vaillancourt JP, Roemer T, Meredith TC. 2011. High-frequency transposition for determining antibacterial mode of action. Nat Chem Biol 7:720-729.
